# An AI-Based Curling Game System for Winter Olympics

**DOI:** 10.34133/2022/9805054

**Published:** 2022-10-20

**Authors:** Xuanke Shi, Quan Wang, Chao Wang, Rui Wang, Longshu Zheng, Chen Qian, Wei Tang

**Affiliations:** ^1^SenseTime Research, Beijing 100080, China; ^2^State Key Laboratory of Fluid Power and Mechatronic Systems, Zhejiang University, Hangzhou 310027, China

## Abstract

The real-time application of artificial intelligence (AI) technologies in sports is a long-standing challenge owing to large spatial sports field, complexity, and uncertainty of real-world environment, etc. Although some AI-based systems have been applied to sporting events such as tennis, basketball, and football, they are replayed after the game rather than applied in real time. Here, we present an AI-based curling game system, termed CurlingHunter, which can display actual trajectories, predicted trajectories, and house regions of curling during the games via a giant screen in curling stadiums and a live streaming media platform on the internet in real time, so as to assist the game, improve the interest of watching game, help athletes train, etc. We provide a complete description of CurlingHunter' architecture and a thorough evaluation of its performances and demonstrate that CurlingHunter possesses remarkable real-time performance (~9.005 ms), high accuracy (30 ± 3 cm under measurement distance > 20 m), and good stability. CurlingHunter is the first, to the best of our knowledge, real-time system that can assist athletes to compete during the games in the history of sports and has been successfully applied in Winter Olympics and Winter Paralympics. Our work highlights the potential of AI-based systems for real-time applications in sports.

## 1. Introduction

Although AI has made a series of breakthroughs in games (such as poker [1] and Go [2]), materials science [3], chemistry [4], biology [5], mathematics [6], debate [7], and ancient text restoration [8], applying AI to sports [9–14] in real time is a challenging problem, because real-time applications in sports to assist games is a domain which requires excellent real-time performance, high accuracy, and good stability, and the environment of sports is a real world that is particularly complex and has many uncertainties, which would greatly affect the performances of the AI-based systems.

The intense curling game at Winter Olympics has attracted great interest and has generated relevant researches [15–17]. As a strategic sport, curling has the reputation of “chess on ice” [18–20]. Its origin could be dated back to the 16th century in Scotland and curling has been an official sporting event of the Winter Olympics since 1998. During the sport, athletes need to pay attention to the positions of the curling stones in real time and make timely strategic adjustments based on information including stones' actual trajectories, predicted trajectories, and house regions. At the same time, these information also significantly influence spectators' feelings of watching games. In addition, analysis of curling stones' motion could provide great help to athletes training and mechanical analysis of curling research. However, in the actual curling game, there is a lack of such real-time system to display these information to assist the games.

Here, we propose an AI-based curling game system, termed CurlingHunter, which can be applied in actual curling games in real time to assist athletes to compete, enhance the interest of the game, etc. Due to the regulations of curling game, no auxiliary equipment can be added to the curling stones; hence, only noncontact measurement methods such as machine vision can be used in CurlingHunter. CurlingHunter has solved these problems: (i) the problem of accurately capturing relatively small curling stones through long-sighted distance (>20 m) in the superlarge space environment with many occlusions; (ii) the problem of lens distortion correction in large scenes without interfering with the ice tracks; (iii) the problem of accuracy of curling stone's visual positioning on the ice sheet; (iv) the problem of occlusions which would interfere with tracking and accuracy, while curling stone is easily blocked by athletes wiping ice, other peoples, or objects during games; (v) the problem of tracking and reidentifying multiple curling stones due to that all curling stones have identical appearance features; and (vi) the problem of runtime in sing-camera system and multicamera system. As the first system to be applied to a curling game, CurlingHunter demonstrated excellent performances in 2022 Beijing Winter Olympic Games of curling and 2022 Beijing Winter Paralympic Games of curling. Although we focus on curling, our system is readily transferable to other sports.

## 2. Results

### 2.1. System Architecture

**Figure 1:**
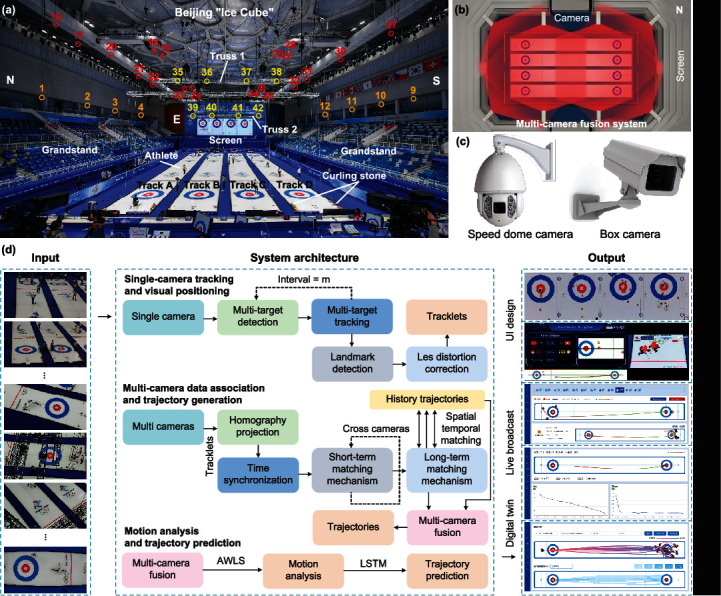
Equipment and system architecture of CurlingHunter. (a) Forty-two cameras were arranged in “Ice Cube” and divided into three heights, i.e., 2nd floor of grandstand (F2), Cat walk (CW), and Truss, where three colors of orange, red, and yellow were used to represent different heights. (b) Schematic diagram of multicamera fusion system with eight cameras. (c) Two types of cameras, i.e., speed dome camera and box camera. Speed dome camera can adjust its angle through its cradle head while box camera cannot move when it is fixed, but its resolution is higher. (d) System architecture. CurlingHunter consists of three main modules: single-camera tracking and visual positioning, multicamera data association and trajectory generation, and motion analysis and trajectory prediction. The input is images taken by each camera, and the output is actual trajectories, predicted trajectories, house regions, motion analysis of curling stones, etc.

The curling game of the 2022 Beijing Winter Olympics was held in Beijing “Ice Cube” (Figure 1(a)), which was the largest curling stadiums in the history of the Olympics. There were four ice tracks about 46-meter length and 5-meter width in the middle of “Ice Cube” (figure S1). Our CurlingHunter consisted of forty-two cameras arranged in “Ice Cube” (Figure 1(a), figure S2, and Materials and Methods) with overlapping field of views (Figure 1(b), figures S3–S6, and Materials and Methods) to ensure that every part of ice tracks was captured by at least three cameras from different angles so as to solve the problem of occlusions including people, truss, and camera. The cameras were arranged on three heights, i.e., 2nd floor of grandstand (F2), Cat walk (CW), and Truss, which were distributed around the ice tracks, including two types with different functions, i.e., speed dome camera and box camera (Figure 1(c)). A large screen with 170 square meters was placed in east side of the stadium to display the house regions and curling stones' actual trajectories and predicted trajectories of the four ice tracks in real time (Figure 1(a)), which could help athletes to make a preliminary judgment and develop a strategy during the game and make watching games more interesting. Two types of curling stones, red and yellow (figure S7), were used in curling game, each with identical appearance.

Due to many uncertainties in practical curling game, it is a huge and complex task to reconstruct curling stones' actual trajectories in real time, predict its future trajectories, and analyze its motion parameters. These processes involve utilizing single-camera tracking, multicamera fusion, lens distortion correction, deep learning, etc. Considering the variety of tasks required to tackle these problems, it seems infeasible to envisage a monolith solution in the form of an end-to-end system, such as a single deep learning network. Instead, CurlingHunter was designed to break these problems into modular tangible tasks. It is interesting that some of these tasks are proposed in this practical curling game, such as optimal tracking strategy for small targets in large environment, visual positioning of targets on the ice, image distortion correction in large scene, and real-time multicamera data association, promoting the proposal and application of some new methods in AI field. In the following, we succinctly introduce all main modules of CurlingHunter and how they handle the information from the previous module. More details of these parts are described in the Materials and Methods.

CurlingHunter consists of three main modules (Figure 1(d)): single-camera tracking and visual positioning, multicamera data association and trajectory generation, and motion analysis and trajectory prediction. The first module is based on single-camera processing, which is multithreaded and executed simultaneously. Through the first module, the data association of multi-frame information of forty-two cameras can be obtained, but the tracklets generated by a single view is easily affected by short-term or long-term occlusions. In the second module, we design a time synchronization to synchronize the tracklets of each camera generated in the first module at the same time, then propose a long short-term matching mechanism (LSTMM) by curling stones' locations and history trajectory information to assign the global curling ID in spatial dimension and match long short-term historical information in time dimension, and lastly, utilize multicamera fusion to reconstruct curling stones' actual trajectories in real time. In the third module, we take advantage of the asymmetric weighted least-square (AWLS) to calculate the velocity, acceleration, and angular velocity of curling stone in real time and propose a model based on the long short-term memory (LSTM) to predict the curling stones' future trajectories in real time.

#### 2.1.1. Single-Camera Tracking and Visual Positioning

**Figure 2:**
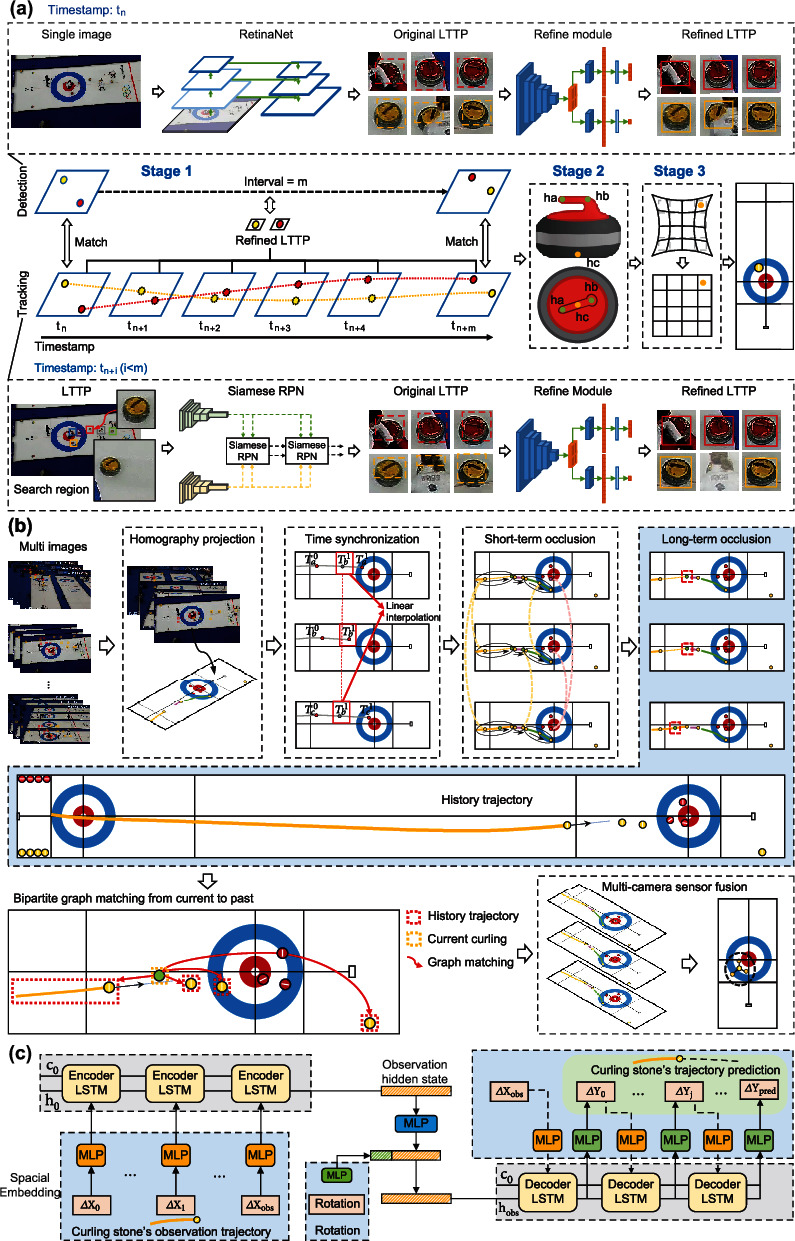
Overview of each module. (a) Overview of single-camera tracking and visual positioning. The module consists of three stages, i.e., sing-camera detection and tracking, landmark detection, and lens distortion correction. (b) Overview of multicamera data association and trajectory generation. The module consists of three components, i.e., homography projection, time synchronization, LSTMM-based region growing, and multicamera fusion module. (c) Overview of trajectory prediction. The framework consists of three components, i.e., encoder, rotation fusion module, and decoder.

Single-camera tracking and visual positioning is achieved through three stages (Figure 2(a)). The first stage is single-camera detection and tracking. Unlike the general tracking-by-detection paradigm, our method mainly focuses on the local target template patch (LTTP), a target-centered image patch which is defined as the smallest carrier of target information that reflects the target appearance features and location information, which can reduce the overhead of a lot of computing resources brought by frame-by-frame detection because a large amount of background information in the image is meaningless for tracking. RetinaNet [21] with multiscale feature pyramid network (FPN) [22] is used to detect curling stones and find their bounding boxes (BBox), so as to obtain the original LTTP. A refine module (details are described in the Materials and Methods) is proposed to update and optimize the original LTTP so as to obtain refined LTTP with high quality. To track the curling stones, we take inspiration from Siamese-RPN [23, 24] and design a lightweight Deep Siamese Tracker (details are described in the Materials and Methods). However, tracking by historical templates cannot meet the need to track new appearing objects in the scene and would be affected by long-term occlusion. We can get new appearing LTTP by the detection module which is called at a certain frequency. The less the detection module is called, the faster the system will be. In order to maximize the performance of the system, our detection module is performed on the whole image every 10 frames, which can achieve good tracking performance and low time overhead. To avoid the exchange of curling ID which has already been tracked, a greedy matching strategy is used to match the currently detected refined LTTP with the historically tracked refined LTTP, and we only use CIoU [25, 26] of the BBoxes of curling in the refined LTTPs to estimate the similarity of curling stones across frames. By combining detection and tracking with refine module, we achieve a robust and efficient tracking process handling complex and changeable real environments, which is suitable for solving the problem of accurately capturing relatively small curling stones through long-sighted distance (>20 m) in the superlarge space environment.

In the second stage, we design a landmark detection network (details are described in the Materials and Methods) to obtain the landmark coordinates of curling stones, so as to get the real position of curling stones in LTTP. Usually, the center of the detection frame is used as the position center of the target, but this method is not suitable for curling stones. For cameras with different viewing angles and different pixel positions of the same camera in a large scene, the perspectives of the curling stone vary greatly, resulting in a completely different positions between the center of the detection frame and the corresponding position of the curling stone. Therefore, if the center of detection frame is used as the position of curling stone, it would cause large systematic errors in global positioning of curling stone. To overcome this problem, we define a curling landmarks consisting of handle head of stone, handle tail of stone, and bottom center of stone, denoted as *h* = (*h*_*a*_, *h*_*b*_, *h*_*c*_) (Figure 2(a)). This method determines position of curling stone as curling landmarks instead of the detection frame center that is time variable, which ensures that curling stone is in the same position from different camera angles, avoiding positioning errors caused by fuzzy definition. We use deep learning to get coordinates of curling landmarks and corresponding accuracy scores.

However, the coordinates of landmarks are affected by image distortion. In the field of machine vision, the distorted image can be rectified by camera calibration [27] using a checkerboard to calibrate the camera, but camera calibration in large scenes is very difficult, and the size and quantity of the checkerboard cannot meet requirements of calibration. Especially in the curling game, the checkerboard cannot be arranged in ice tracks due to that the ice tracks would be interfered by checkerboard. In this stage, to solve the problem of checkerboard method failure, we take examples from algebraic methods [28, 29] and propose a fully automatic lens distortion correction method (figure S8 and Materials and Methods), which is based on the structured straight-line elements in images. The method constructs an appropriate energy function for the linear elements in the image and utilizes nonlinear optimization to iteratively correct the distorted linear elements, so as to complete lens distortion correction. By the method, we do not need to calibrate intrinsic parameters of cameras and can easily get the distortion correction model through only a single image in a large scene. Through the distortion correction model, we can easily obtain accurate coordinates of curling stones.

#### 2.1.2. Multicamera Data Association and Trajectory Generation

All curling information tracked by the single-camera system is fed into the multicamera system, as shown in Figure 2(b), and the position of curling stone in the world coordinate system is a bridge for different cameras. To take advantage of the complementary gains of each camera, we use the world coordinates of curling stones to integrate the information of each camera. The coordinates of curling stones in the image of each camera can be projected on the ice tracks by using proposed homography projection (details are described in the Materials and Methods), so as to complete the transformation from the image coordinate system to the world coordinate system. The world coordinates of curling stones are stored in the queue of each camera. In order to avoid the time asynchronous interference and simultaneously obtain the curling position information of each camera, we adopt a time synchronization algorithm (details are described in the Materials and Methods) to complete the timestamp alignment of each camera. And then the time-aligned curling stones' information of each camera at the reference timestamp is obtained, which is the base to associate the tracklet information from different cameras.

There are two challenges in multicamera tracking: one is that each camera only covers a part of the curling stones, resulting that the total number of curling stones is difficult to determine and the total number that can be seen from multiple cameras may vary in the time dimension; the other is that it is hard to associate the information across different cameras due to that all curling stones have identical appearance features. In addition, it is a well-known NP-hard problem to tackle the data association problem across cameras, and the runtime of the algorithm grows exponentially as the number of cameras grows; existing methods are mostly offline and cannot meet the requirements of real-time data processing. A long short-term matching mechanism (LSTMM) (table S1 and Materials and Methods) based on regional growing algorithm is proposed to assign the global curling ID in spatial dimension and match long-short term historical information in time dimension to overcome these problems. With the help of accurate spatial temporal curling positioning and the local appearance information from tracklets, the problem of frequent ID switch in single-camera tracking caused by short-term occlusion can be solved.

In some cases, the curling stone cannot be captured and tracked by any camera due to occlusion or other reasons. However, the reidentification technique cannot be used since it would mislead the matching process due to that all curling stones have identical appearance features. To enhance performance of multicamera tracking in long-term occlusion, a global spatial temporal matching mechanism (details are described in the Materials and Methods) by bigraph matching is proposed. With the long-term matching mechanism, the region growing algorithm can be executed along the time dimension and curling stones which have been occluded for a long time can find the corresponding ID. Curling stones are merged from different cameras which have a same global curling ID. To improve the accuracy of multicamera sensor fusion, we use the accuracy confidence scores as a guide for weighted fusion of curling coordinates. Finally, global trajectories of curling stones can be generated in real time by multicamera sensor fusion.

#### 2.1.3. Motion Analysis and Trajectory Prediction

The kinematic and mechanical analysis of curling [15, 30] is difficult and complex. The characteristics of the ice surface would be affected by a series of factors, such as temperature, humidity, and athletes rubbing the ice, resulting in some local or overall small changes in the ice surface, which would even affect the final results of curling games. High-quality curling motion data is of great significance for the mechanical analysis of curling motion and the study of ice surface characteristics. The asymmetric weighted least square method (figure S9 and Materials and Methods) combined with high-frequency motion capture based on forty-two cameras is used to calculate the velocity, acceleration, and angular velocity of curling stone in real time, which can reflect the quality of the ice surface and help athlete train.

Uncertainty in the overall and local behavior of the ice sheet have brought great challenges to modeling curling motion, and the frictional force of the ice changes essentially with each throw. In addition to the uncertainty of the ice surface, the ice rubbing by athletes and multicamera measurement errors are also difficult to model by physical models. All of these factors make the precise future state of curling intrinsically unpredictable. Although the motion modeling of curling contains many uncertainties, the motion behaviors of curling in the future can be approximated. The motion of curling approximately satisfies the Markov assumption [31], so we can adopt sequence model to predict the curling stone's future trajectory. We introduce an encoder-decoder framework (details are described in the Materials and Methods) based on long short-term memory (LSTM) network [32] which predicts the future trajectory based on curling's partial observation in a throw. The framework of our trajectory prediction model is shown in Figure 2(c), which consists of three key components: encoder, rotation fusion module, and decoder. Through the model, CurlingHunter can obtain curling stone's predicted trajectory in real time, which can help athletes judge and enhance the interest of the game.

### 2.2. Evaluation and Applications

To comprehensively evaluate the performances of CurlingHunter, we conducted detailed experiments and applied it in actual curling games. First of all, we tested the effectiveness and positioning accuracy of each module, evaluated the overall real-time performance, and compared with the existing AI systems used in sport games to verify that only CurlingHunter could be applied in real time during the games and be broadcast live, while other existing AI systems could not achieve. Finally, we presented the applications of CurlingHunter in actual curling games, including 2021 Wheelchair Curling World Championships, 2022 Beijing Winter Olympics, and 2022 Beijing Winter Paralympics.

#### 2.2.1. System Evaluation

**Figure 3:**
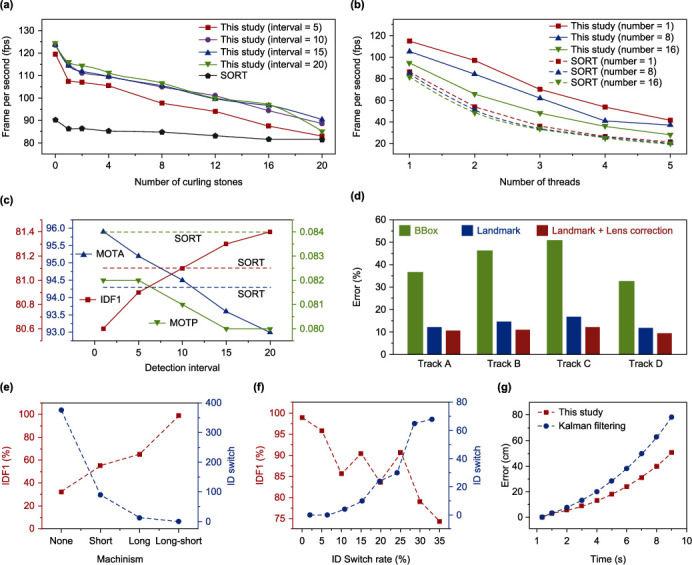
Evaluation of CurlingHunter. (a) The relationship of tracking speed (fps) versus number of curling stones. (b) The relationship of tracking speed (fps) versus number of thread for a shared Telsla T4 GPU. (c) The relationship of IDF1, MOTA, and MOTP versus detection interval. (d) Comparison of the center coordinate of BBox, landmark detection, and landmark detection with lens distortion correction in visual positioning error. (e) Comparison of without any mechanism, short-term matching mechanism, long-term matching mechanism, and long short-term matching mechanism in IDF1 and ID switch. (f) The relationship of IDF1 and ID switch versus ID switch rate in long short-term matching mechanism. (g) Comparison of our method and Kalman filtering. The error of curling stone's trajectory prediction mainly comes from measurement error of observation trajectory and uneven distribution state of ice surface.

To verify the effectiveness of single-camera tracking and minimize the time and resource overhead of single-camera tracking, we conducted relevant verification based on the actual situation. For quantitative evaluation, we adopted IDF1, MOTA, and MOTP for tracking performance which was widely accepted by CLEAR MOT metrics [33]. At the same time, we used FPS (frame per second) to measure the time overhead of the program. As shown in Figure 3(a), the runtime of our method increases gradually with the number of targets. Nonetheless, our method always outperforms frame-by-frame detection schemes (such as SORT), since the image contains a large number of invalid regions by using frame-by-frame detection schemes. In addition to the time advantage, our method can bring a smaller resource overhead, since using one graphics card per video is a luxury in practical applications. As shown in Figure 3(b), the frame-by-frame detection scheme (such as SORT) produces little changes with the increase of the number of objects in the scene, because the detection is carried out on the whole image. As the number of processed videos increases, it is difficult to guarantee real-time performance with limited resources when using the full-image tracking scheme. However, our method only using the search region near the target template for tracking task is better than frame-by-frame detection, which is instructive for the deployment of tracking algorithms with limited resources. As shown in Figure 3(c), as the detection interval increases, the IDF1 of our method becomes better, MOTA becomes worse, and MOTP becomes better. To strike a balance between performance and time, we set the detection interval to ten, in which case our method performs better than SORT algorithm that does not use the appearance feature of target. To verify the validity of our refine module, we designed an ablation experiment. As shown in table S2, the high-quality target template refined by refine module help us to get better performance than original SiamsRPN++. To verify the accuracy of our visual positioning, we evaluated the measurement accuracy of five points at base camp with known coordinates for each ice track. As shown in Figure 3(d), the proposed landmark detection has a large accuracy improvement over the center coordinate of BBox, and the error is further reduced by lens distortion correction, thereby further improving the accuracy and achieving 30 ± 3 cm under measurement distance > 20 m. Movie S1 restores the actual trajectory to the ice track so as to visually verify the accuracy of CurlingHunter. High accuracy guarantees that CurlingHunter can be used in actual curling games.

We used IDF1 and ID-Switch to quantitatively evaluate the performance of multicamera tracking to reconstruct trajectories and analyzed the effects of long-term matching mechanism, short-term matching mechanism, and single-camera tracking performance of each camera on multicamera tracking and reconstruction of trajectories, respectively. The ablation experiments were conducted on the multiview videos of a curling game, where 12 videos covered a complete track and lasted about 15 minutes. The ID switch rate could reflect bad case in tracking caused by occlusion or other reasons. As shown in Figure 3(e), long short-term matching mechanism is the best in both metrics. As shown in Figure 3(f), we actively introduce per-camera ID switch probabilities ranging from 0% to 35%, although the probability of per-camera local ID switch is large enough, we find that the global ID switch is still relatively small. Our method is robust in complex and varied real-world environments due to that we combine single-camera tracking information and curling motion information for long-term short-term matching rather than relying only on a single submodule.

We evaluated the results of velocity and angle measurements in a wheelchair curling game where no athletes rubbed the ice. As shown in figure S10, the noise of velocity and angle calculation due to measurement error can be eliminated in real time, so we can obtain the monitoring of the motion information of curling during the curling movement without attaching any additional equipment. As shown in Figure 3(g), our method predicts future trajectories better than those estimated by Kalman filtering.

To verify the real-time performance of CurlingHunter, we conducted an overall evaluation of the runtime of each module (tables S3–S4, and Materials and Methods). Unlike the usual researchers discussing a specific method, we focus on how to comprehensively utilize each module to realize that the whole performance better than parts. We tested the overall runtime of CurlingHunter in a large number of actual curling games, and the average runtime is ~9.005 ms, the time lag of which human eyes cannot distinguish, demonstrating that CurlingHunter can be applied for actual curling games in real time.

Existing AI systems are used in tennis [9], basketball [10], and football [11], which are mainly used for postgame analysis to help athletes train or assist the referee in judging the games, and cannot be applied in real time to assist games. The Hawk-Eye System used in tennis is the most mature and advanced technologies applied in sports, but its runtime is ~10 s, which is above 1,000 times slower than ours (our CurlingHunter only takes ~9.005 ms). Table S5 compares CurlingHunter with existing AI systems in detail, demonstrating that CurlingHunter is the first AI sports system in history that can be applied in real time to assist the game and improve the interest of watching game, etc.

#### 2.2.2. Winter Olympics

We conducted many practical tests on CurlingHunter, where movie S2 shows one of the tests, and invited relevant professionals to evaluate the results. The results show that CurlingHunter possesses remarkable real-time performance (~9.005 ms), high accuracy (30 ± 3 cm under measurement distance > 20 m), and good stability, proving that CurlingHunter can be used in actual curling games. We applied CurlingHunter to the 2021 Wheelchair Curling World Championships from October 23rd, 2021 to October 30th, 2021, 2022 Beijing Winter Olympic Games of curling from February 10th, 2022 to February 17th, 2022, and 2022 Beijing Winter Paralympic Games of curling from March 5th, 2022 to March 15th, 2022, and achieved excellent results, which obtained highly commended from the World Curling Federation (WCF) President Kate Caithness, athletes, coaches, referees, spectators, commentators, media, etc.

**Figure 4:**
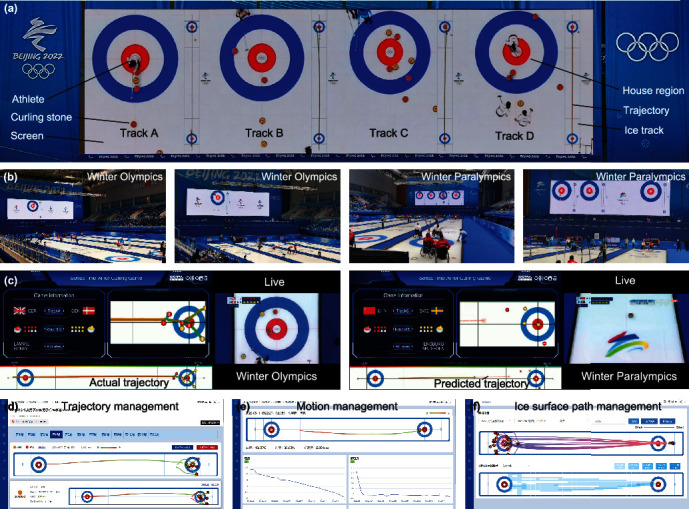
2022 Beijing Winter Olympics Games and 2022 Beijing Winter Paralympics. (a) The giant screen with an area of 170 square meters. (b) The actual applications of CurlingHunter in Winter Olympics and Winter Paralympics. (c) The live video streaming of CurlingHunter in Winter Olympics and Winter Paralympics. (d) The trajectory management system. (e) The motion analysis management system. (f) The ice surface path management system.

The giant screen with an area of 170 square meters, as shown in Figure 4(a), displays the four house regions and the curling stones' trajectories in real time at a 1 : 1 ratio. Figure 4(b) shows the actual applications of CurlingHunter in Winter Olympics and Winter Paralympics, where athletes “watching the giant screen” during the games has become the norm. The curling ice tracks are very long, resulting that athletes are dozens of meters away from the house regions, so it is difficult to know where the target is located dozens of meters away and how close to the center of the house region in the past. In addition, in the past, athletes could only rely on memory for the trajectory of each throw, and how to correct the next throw could also only depend on memory. CurlingHunter solves these problems technically. By watching the giant screen, athletes could clearly know the actual positioning of curling stones, the actual trajectory, the predicted trajectory, the specifics of the current throw, and how to correct the next throw, which greatly liberate the memory of athletes, so as to better assist athletes in curling games.

Through the live video streaming of CurlingHunter (Figure 4(c), movie S3, and movie S4), the spectators can clearly see the trajectory of each throw by the athletes. In the past, the spectators could only watch a partial perspective of the live broadcast and did not know the curling in other three ice tracks or the overall situation of the curling game, but CurlingHunter presents to spectators the most intuitive and comprehensive display, significantly enhancing the experience of watching the games. Figure S11 and movie S5 show curling stone's velocity, acceleration and rotation angle in real time during the games. In addition, we developed a management system (Figures 4(d)–4(f) and figure S12) for CurlingHunter to record and manage the trajectory, motion analysis, and ice surface path for each curling game. The management system can save all the game matches and their related information, including the team of the game, the person who threw the curling stone, the direction of the game, time, temperature, and humidity. The trajectory management system (Figure 4(d)) can dynamically display the trajectory of the curling stone; the motion management system (Figure 4(e)) can dynamically display the velocity, acceleration, and rotation angle of the curling stone; and the ice surface path management system (Figure 4(f)) can dynamically display the friction degree of the ice surface during the curling movement. The management system can be used for game management, postmatch analysis, and postmatch training for athletes, etc. Interestingly, the actual motion data of the curling games can be used for mechanical analysis of curling research.

## 3. Discussion

In this work we developed CurlingHunter, a curling game system based on a series of AI technologies, with remarkable real-time performance (~9.005 ms), high accuracy (30 ± 3 cm under measurement distance > 20 m), and good stability. CurlingHunter has been successfully applied to actual curling game, filling in the gaps of the systems which are utilized to assist curling game in real time. CurlingHunter is the first, to the best of our knowledge, real-time system that assist athletes to compete during the games in the history of sports and successfully applied in Winter Olympics and Winter Paralympics. The achievements described in this work represent a major milestone in the development of AI technologies applied in the real world and promote the development of curling games. In addition, CurlingHunter offers a new platform for further extending to other sports and using to academic research of multi-target multi-camera tracking.

## 4. Materials and Methods

### 4.1. Curling Game

Curling, as a combination of bowling and chess [31], is a turn-based game in which two teams play alternately on the ice tracks. There are four ice tracks in curling game, where each ice track consists of side line, house region, hack, tee line, and hog line, as shown in figure S1. There are eight athletes in the two teams, and there are usually ten round games. A curling game requires two sets of curling stones, where each set consists of eight curling stones. Different from the ice surface of figure skating or short track speed skating, the ice track surface of curling game is not completely flat, whose top layer is covered with specially made tiny particles; hence, athletes need to sweep the ice surface to change the friction between the curling stone and the ice surface so as to adjust the direction. As show in figure S7, the diameter, height, and weight of curling stone are 30 cm, 11.43 cm, and 19.96 kg, respectively.

### 4.2. Positions and Layouts of Cameras

The forty-two cameras are divided into three heights, i.e., 2nd floor of grandstand (F2), Cat walk (CW), and Truss, which are distributed around the ice tracks (Figure 1(a) and figure S2A). The cameras consist of 12 box cameras and 30 speed dome cameras, where the F2 is box cameras and the others are speed dome cameras. A speed dome camera can adjust its angle through its cradle head while box camera cannot move when it is fixed, but its resolution is higher. There are 12 box cameras arranged in F2 (figure S2B), that is north side of F2 (F2: north grandstand): cameras 1-4, south side of F2 (F2: south grandstand): cameras 9-12, and west side of F2 (F2: LED): cameras 5-8. There are 22 speed dome cameras arranged in CW (figure S2C) that is north 1 of CW (CW: north 1): cameras 16, 17, 20-22; north 2 of CW (CW: north 2): Cameras 15, 18, 19, 23, 24; south 1 of CW (CW: south 1): cameras 26, 27, 30-32; south 2 of CW (CW: south 2): cameras 25, 28, 29, 33, 34; and west of CW (CW: west): cameras 13, 14. There are 8 speed dome cameras arranged in CW (figure S2D), that is, west of truss (Truss 1: west house region): cameras 35-38 and east of truss (Truss 2: east house region): cameras 39-42.

### 4.3. Coverage Areas of Cameras

The forty-two cameras overlap and cover all areas of the ice tracks, ensuring that every part of the ice tracks are covered by at least three cameras with different viewing angles to solve the problems of occlusion. Each camera has a jurisdictional area, and the actual monitor area of each camera is larger than its jurisdictional area. Cameras 1-4 and 9-12 in F2 overlap each other and cover two tracks in the north and south, respectively, ensuring that every part of the ice tracks is covered, where their jurisdictional areas are shown in figure S3A and actual monitor areas are shown in figure S4. Cameras 5-8 in F2 overlap each other and cover far and near ends of two tracks in the north and south, respectively, ensuring that every part of the ice tracks are covered, where their jurisdictional areas are shown in figure S3B and actual monitor areas are shown in figure S4. Cameras 16, 17, 20-22 and 26, 27, 30-32 in CW overlap each other and cover the first track in the north and south, respectively, ensuring that every part of the ice tracks A and D is covered, where their jurisdictional areas are shown in figure S3C and actual monitor areas are shown in figure S5. Cameras 15, 18, 19, 23, 24 and 25, 28, 29, 33, 34 in CW overlap each other and cover the second track in the north and south, respectively, ensuring that every part of the ice tracks B and C is covered, where their jurisdictional areas are shown in figure S3D and actual monitor areas are shown in figure S5. Cameras 13, 14 in CW cover near ends of two tracks in the north and south, respectively, where their jurisdictional areas are shown in figure S3E and actual monitor areas are shown in figure S5. Cameras 35-42 cover 8 house region, respectively, where their jurisdictional areas are shown in figure S3F and actual monitor areas are shown in figure S6.

### 4.4. Single-Camera Tracking and Visual Positioning

#### 4.4.1. Detection

For generation of the initial LTTP, RetinaNet [21] with multiscale feature pyramid network (FPN) [22] is used to detect curling stones and find their bounding boxes (BBox). FPN adopts top-down architecture with skip connections, which can produce a single high-level feature map with fine resolution. We detect the curling stones on the finest layer combining the high-level and low-level semantics, which is useful for accurate localization of small curling stones as it has less secondary sampling of the original image. However, there are a lot of problems in the original LTTP generated by detection, such as exiting false positives, jitter of the curling stone' bounding boxes, and poor quality of the local target template patch. To solve these problems, a refine module is proposed to update and optimize LTTP.

#### 4.4.2. Refine Module

The refine module consists of two branches, regression branch and confidence branch, which are used to optimize the coordinate of the original LTTP in the image and estimate the quality of final LTTP. For original LTTP, if LTTP slightly deviate from the ground truth, the LTTP coordinate in the image could be adjusted by regression branch; if LTTP greatly deviate from the curling or does not contain any curling stone by confidence branch, the LTTP could be discarded.

The training of the refine module consists of two stages. Firstly, we train the regression branch end to end using stochastic gradient descent (SGD) with momentum. During the training, we augment the data by applying random occlude, rotate, horizontal flip, and so on, which can significantly enhance the generalization and robustness of the neural network for complex scenes. To make the regression easier to converge, we make the proportion of curling fixed as *α* = 1.2 and normalize LTTP as same size. The loss for regression is smooth *L*_1_ loss. The center coordinate of LTTP is (*x*_*c*_, *y*_*c*_), and the refine bounding box in LTTP is (*w*_*d*_, *h*_*d*_). We can directly regress the values of (*δ*_1_, *δ*_2_, *δ*_3_, *δ*_4_) to rectify the coordinate in the original patch (*x*_*c*_, *y*_*c*_, *w*_*d*_, *h*_*d*_) due to that the patch is normalized. Ground truth in LTTP can be denoted as (*g*_1_, *g*_2_, *g*_3_, *g*_4_). The smooth *L*_1_ loss is

(1) $$\begin{array}{c} {\text{smooth}}_{L_{1}}\left(x,\sigma \right)=f\left(x\right)=\left\{\begin{array}{cc} \frac{1}{2}\sigma^{2}x^{2}, & \left|x\right|\leq \frac{1}{\sigma^{2}},\\ \left|x\right|-\frac{1}{2\sigma^{2}}, & \;\left|x\right|>\frac{1}{\sigma^{2}}. \end{array}\right. \end{array}$$

The regression loss is

(2) $$\begin{array}{c} L_{\text{reg}}=\overset{3}{\underset{i=0}{\sum }}{\text{smooth}}_{L_{1}}\left(\delta_{i}-g_{i},\sigma \right). \end{array}$$

Secondly, the confidence branch shares the same feature extraction subnetwork with regression branch. We freeze the weighted value of the regression branch when it converges. To quantify the quality of LTTP more accurately and remove the false positive detection, we directly regress the confidence score of LTTP. The quality score of LTTP can be approximated truncated CIoU [25, 26] between bounding boxes from detection result *D* and result $\hat{D}$ from the regression branch. To balance the positive and negative samples, we collected a large amount of low quality LTTP and set its score to zero. The truncated CIoU is

(3) $$\begin{array}{c} \text{confidence}\left(D\right)=\left\{\begin{array}{cc} CIoU\left(D,\hat{D}\right), & CIoU\left(D,\hat{D}\right)>0,\\ 0, & CIoU\left(D,\hat{D}\right)\leq 0, \end{array}\right. \end{array}$$

where confidence(*D*) ranges continuously from 0 to 1.

During the inference phase, the two branches simultaneously output the refined bounding boxes and the quality score of LTTP. The low-quality LTTP or false detection would be removed if the confidence score is less than thr_low_. Finally, the quality of LTTP is improved by the refine module.

#### 4.4.3. Tracking

The proposed lightweight Deep Siamese Tracker consists of a Siamese subnetwork for feature extraction and a region proposal subnetwork for proposal generation. For feature extraction, the template branch encodes the historical target patch improved by proposed the refine module, the detect branch encodes the search patch which contains the region in current frame where the target patch in previous frame was located. For region proposal, the template feature and the search patch feature can be associated by a correlation operation. Then, the region proposal network [34] is adopted to regress the coordinate of the target proposal and finish the foreground-background classification. We get new LTTP in next frame after tracking, to improve the quality of LTTP, the refine module is used to improve the LTTP and estimate its confidence score which can remove the illegal LTTP. However, the number of objects in the scene changes dynamically, and new target may appear in the field of view at any time; we can get new appearing LTTP by the detection module which is called at a certain frequency as a supplement. To avoid the exchange of curling ID which has already been tracked, a greedy matching strategy is used to match the currently detected refined LTTP with the historically tracked refined LTTP. We only use CIoU [25, 26] of the BBoxes of curling in the refined LTTPs to estimate the similarity of curling stones across frames because all the curling stones with same color have an identical appearance feature. When the similarity is greater than 0.5, the ID of the curling keep the same as the historical tracking information; otherwise, we assign the tracking ID to the newly appearing curling. By the skillful combination of detection and tracking with the refine module, we achieve a robust and efficient tracking process handling complex and changeable real environments which is suitable for tracking multiple small targets in large scenes.

#### 4.4.4. Visual Positioning

The landmark detection is processed on the refined LTTP. As shown in Figure 2(a), we define curling landmarks which consist of landmarks of stone handle head, stone handle tail, and stone bottom center, denoted as *h* = (*h*_*a*_, *h*_*b*_, *h*_*c*_). When the rectified curling proposal is given, we detect the landmarks of curling to accurately improve accuracy of visual measurement. The network architecture of curling landmark detection is similar to the refine module, so we directly regress the coordinate of curling landmark in the normalized LTTP, and the landmark accuracy score is also given by the confidence branch. For each landmark$\;{\hat{h}}_{i}\in h$, the landmark predict accuracy score is defined as

(4) $$\begin{array}{c} \text{confidence}\left({\hat{h}}_{i}\right)=\left\{\begin{array}{cc} 1-\frac{\sigma \left(h_{i},{\hat{h}}_{i}\right)}{{\hat{h}}_{a}-{\hat{h}}_{b}}, & \frac{\sigma \left(h_{i},{\hat{h}}_{i}\right)}{{\hat{h}}_{a}-{\hat{h}}_{b}}\leq 1,\\ 0, & \frac{\sigma \left(h_{i},{\hat{h}}_{i}\right)}{{\hat{h}}_{a}-{\hat{h}}_{b}}>1, \end{array}\right. \end{array}$$

where ${\hat{h}}_{i}$ is the predict landmark and *h*_*i*_ is the label of the ground truth. The accuracy score is normalized by the predict handle length $\left\|{\hat{h}}_{a}-{\hat{h}}_{b}\right\|$, which distributes between 0 and 1. The more accurate the landmark regression accuracy is, the closer the confidence score is to 1; otherwise, it is closer to 0. When the confidence score is greater than 0.75, the system is optimum. Usually, we find the position landmark is more robust than handle landmark due to that the former is the overall feature of curling stone while the latter is the local feature of curling which is easily affected by occlusion. With the accuracy score for each curling in each camera, the problem of landmarks' inaccurate estimation could be improved due to occlusion or other reasons.

#### 4.4.5. Lens Distortion Correction

The distortion model can be defined as

(5) $$\begin{array}{c} \left(\begin{array}{c} x-x_{c}\\ y-y_{c} \end{array}\right)=L\left(r\right)\left(\begin{array}{c} x_{d}-x_{c}\\ y_{d}-y_{c} \end{array}\right), \end{array}$$

where (*x*, *y*) is the coordinate in the corrected image, (*x*_*d*_, *y*_*d*_) is the coordinate in the original image, and (*x*_*c*_, *y*_*c*_) is the center of the lens distortion model. We define *r* as the distance from image point to the center of the lens distortion model. It can be calculated as

(6) $$\begin{array}{c} r=\sqrt{{\left(x_{d}-x_{c}\right)}^{2}+{\left(y_{d}-y_{c}\right)}^{2}.} \end{array}$$

*L*(*r*) determines the distribution of the image distortion which is given by

(7) $$\begin{array}{c} L\left(r\right)=k_{0}+k_{1}r^{2}+k_{2}r^{4}. \end{array}$$

A fully automatic lens distortion correction method is proposed. Firstly, we adopt the improved Hough Line Transform [28] to detect the distorted lines in the image. We denote **k**_**c**_ by the tuple (*k*_0_, *k*_1_, *k*_2_, *x*_*c*_, *y*_*c*_) which defines the distortion model. The undistorted image point *x*_*ij*_ is *j*^th^ point of line *i* in the undistorted image by using equation (5) to rectify the original image point. By distortion model **k**_**c**_, the line *j* in the undistorted image is formulated as

(8) $$\begin{array}{c} \cos\left(\alpha_{i}^{k_{c}}\right)x+\sin\left(\alpha_{i}^{k_{c}}\right)y+d_{i}^{k_{c}}=0. \end{array}$$

Then, an iterative nonlinear optimization is performed by minimizing the average of the square distance from the corrected image points to the corresponding line. The energy function is given by

(9) $$\begin{array}{c} E\left(k_{c}\right)=\frac{\sum_{i=1}^{N_{i}}\sum_{j=1}^{N_{ij}}\left(\cos\left(\alpha_{i}^{k_{c}}\right)x_{ij}+\sin\left(\alpha_{i}^{k_{c}}\right)y_{ij}+d_{i}^{k_{c}}\right)}{\sum_{i=1}^{N_{i}}N_{ij}}. \end{array}$$

We fix the center point of lens distortion (*x*_*c*_, *y*_*c*_) and adopt Levenberg-Marquardt algorithm to minimize the energy function as below:

(10) $$\begin{array}{c} k_{c}^{n+1}=k_{c}^{n}-{\left(\nabla^{2}\text{E}\left(k_{c}^{n}\right)+\lambda I\right)}^{-1}\nabla E\left(k_{c}^{n}\right). \end{array}$$

By the automatic lens distortion correction method, we do not need to calibrate intrinsic parameters of cameras and can easily get the distortion correction model through only single image in a large scene. And the curling landmarks could be corrected by using equation (5). By this method, accurate visual positioning in large scenes becomes feasible, which is important for cross-camera association.

### 4.5. Multicamera Data Association and Trajectory Generation

#### 4.5.1. Homography Projection

For *N* cameras with different views, we use *L*_*t*_ to denote the tracking and positioning results in the same batch of video frames received at time *t*:

(11) $$\begin{array}{c} L_{t}=\left\{\left(L_{t}^{1},T_{t}^{1}\right),\left(L_{t}^{2},T_{t}^{2}\right),\cdots ,\left(L_{t}^{N},T_{t}^{N}\right)\right\}, \end{array}$$

where (*L*_*t*_^*i*^, *T*_*t*_^*i*^) represents the information of *i*^th^ camera at time *T*_*t*_^*i*^. For each tracklet in *L*_*t*_^*i*^ ∈ *L*_*t*_, *i* = 1, ⋯, *N*. We use homography matrix *H*_*i*_ to transform the coordinate from image plane of *N*^th^ camera to the world plane. As shown in Figure 2(b), the curling trajectories of each camera are generated synchronously by homography projection. We use *G*_*t*_ to denote the same batch trajectories of all the cameras:

(12) $$\begin{array}{c} G_{t}=\left\{\left(G_{t}^{1},T_{t}^{1}\right),\left(G_{t}^{2},T_{t}^{2}\right),\cdots ,\left(G_{t}^{N},T_{t}^{N}\right)\right\}, \end{array}$$

where *G*_*t*_^*i*^ contains all the curling trajectories of *i*^th^ camera and *T*_t_^*i*^ represents the corresponding timestamp.

#### 4.5.2. Time Synchronization

The timestamps from different cameras are not strictly aligned. For the timestamps *T*_*t*_^*i*^ and *T*_*t*_^*j*^, *i*, *j* = 1, ⋯, *N*, *i* ≠ *j*, they are usually not equal numerically. In some extreme cases, they could also be significantly out of sync. Therefore, we store the trajectory data of different cameras in their respective queue data structures, and use the queue head data of different cameras as the same batch *G*_*t*_. When the biggest timestamp interval is larger than the *T*_thr_, we dequeue the trajectory data with the smallest timestamp and enqueue the data of the next frame until the same batch of data satisfies that the timestamp interval is less than the *T*_thr_, where *T*_thr_ = 33 ms.

To avoid the time asynchronous interference of each camera, we linearly interpolate the coordinates of the curling along the time dimension. As shown in Figure 2(b), *T*_*t*+1_^2^ is the earliest timestamp in the same batch. We use linear interpolation for trajectories of other cameras to get the trajectory information at time *T*_*t*+1_^2^. It can be calculated as

(13) $$\begin{array}{c} G_{t_{1}}^{i}=G_{t_{0}}^{i}\times \frac{t_{1}-t}{t_{1}-t_{0}\;}+G_{t_{1}}^{i}\times \frac{t-t_{0}}{t_{1}-t_{0}}, \end{array}$$

where *t* is the timestamp to be interpolated. $\tilde{G_{t}}$ is used to denote the aligned local trajectories:

(14) $$\begin{array}{c} \tilde{G_{t}}=\left\{\left({\tilde{G}}_{t}^{1},{\tilde{G}}_{t}^{2},\cdots ,{\tilde{G}}_{t}^{N}\right),\tilde{T_{t}}\right\}, \end{array}$$

where ${\tilde{G}}_{t}^{i}$ is the curling trajectories of i-th camera at aligned timestamp $\tilde{T_{t}}$.

With the time synchronization algorithm, the time-aligned curling stones' information of each camera at the reference timestamp is obtained, which is the base to associate the tracklet information from different camera.

#### 4.5.3. Multicamera Tracking

The proposed LSTMM is outlined in table S1. For a single view, the tracklet can pass the GCID information to the next frame. We denote the initial curling seeds by set *S*_*t*_ which collects the local spatial temporal association from all cameras at *T*_*t*_ timestamp. We select the initial seeds which have been assigned GCID by heuristic information of each camera tracking result. For each curling $c_{t}^{ij}\in {\tilde{G}}_{t}^{i}$, we compute the pairwise euclidean distance between the curling *c*_*t*_^*j*^ without GCID and the seed curling *c*_*t*_^*k*^ belongs to initial seeds set *S*_*t*_. The growth criteria is as follows:

(15) $$\begin{array}{c} {\left\|c_{t}^{ij}-c_{t}^{k}\right\|}_{2}<\text{dis}{\text{t}}_{\text{thr}},j=1,\cdots ,M_{i}k=1,\cdots ,K, \end{array}$$

where *M*_*i*_ is the total number of curling stones in the aligned trajectory ${\tilde{G}}_{t}^{i}$, *K* is the total number of initial seeds in set *S*_*t*_, and dist_thr_ = 30 cm which is equal to the diameter of a curling stone. As shown in Figure 2(b), when the condition is satisfied, the same batch curling stones from different views should be gradually clustered to be a same GCID identity in the world plane, until the same batch of curling stone from different views is traversed. With the help of accurate spatial temporal curling positioning and the local appearance information from tracklet, the problem of frequent ID switch in single-camera tracking caused by short-term occlusion can be solved.

Most of the curling stones in the cameras have been assigned a GCID through the single-camera tracking and the short-term matching mechanism. However, owing to the complexity of the real-world environment, the curling identities are often occluded by the objects, e.g., athletes, trusses in the air. We design a spatial temporal long-term matching mechanism to compensate for the limitations of short-term matching. We construct a bipartite graph between the historical trajectories and the curling stones without GCID for each view. To reduce the solution space of this problem, we design an elimination criteria to exclude solutions which are impossible. Firstly, for each camera, we remove the trajectories where GCID have been assigned in this view. Secondly, we remove the impossible matching from current to history trajectory. To improve the reuse rate of the latest state of the trajectory, we estimate the curling stones' motion state and trajectory tangent equation of each trajectory at the latest moment. For a certain curling stones' trajectory at *T*_*t*_ timestamp, we choose the *Q* stones' coordinate in the trajectory which are closest to time *T*_*t*_, where the stones' coordinate at time *T*_*t*_ is *c*_*t*_ = (*x*_*t*_, *y*_*t*_). The trajectory can be represented by a quadratic equation as follows:

(16) $$\begin{array}{c} x=a_{1}y^{2}+a_{2}y+a_{3}, \end{array}$$

where *a*_1_, *a*_2_, and *a*_3_ are the coefficients of the equation. For this least squares problem **X** = **AY**. We use normal equation to get the optimal solution.

(17) $$\begin{array}{c} A={\left(Y^{T}\;Y\right)}^{-1}Y^{T}X. \end{array}$$

By the trajectory equation, the trajectory tangent equation at (*x*_*t*_, *y*_*t*_) is

(18) $$\begin{array}{c} k_{1}x+k_{2}y+k_{3}=0, \end{array}$$

where *k*_1_ = 1, *k*_2_ = −2*a*_1_*y*_*t*_ + *a*_2_, and *k*_3_ = *k*_2_*y*_*t*_ − *x*_*t*_. In order to determine whether the curling stone is in a stationary state. We construct a curling stone's motion status matrix *S* from the latest *Q* zero-centered curling stone's coordinates. By singular value decomposition (SVD), we have **S** = **U***Σ ***V**^*T*^, where the singular value are *σ*_1_ and *σ*_2_ which can reflect the variance of the curling stone's coordinate in the direction of columns vector of **V**^*T*^. When the singular value *σ*_1_ and *σ*_2_ are smaller than *σ*_thr_, we set *a*_1_ = *a*_2_ = *a*_3_ = 0 to represent the curling stone is stationary in the past. Based on the above formula, the second elimination criteria is as follows:

(19) $$\begin{array}{c} \left\{\begin{array}{cc} {\left\|c_{t+1}-c_{t}\right\|}_{2}<dis{\text{t}}_{\text{thr}}, & \text{if }a_{1}=a_{2}=a_{3}=0,\\ \frac{\left|k_{1}x_{t+1}+k_{2}y_{t+1}+k_{3}\right|}{\sqrt{k_{1}^{2}+k_{2}^{2}}}<dis{\text{t}}_{\text{thr}}, & \text{if not }{\text{a}}_{1}=a_{2}=a_{3}=0,\\ \frac{{\left\|c_{t+1}-c_{t}\right\|}_{2}}{T_{t+1}-T_{t}}<v_{\text{thr}}. \end{array}\right. \end{array}$$

For each curling stone $c_{t+1}^{j}\in {\tilde{G}}_{t+1}^{i}$, if the *j*^th^ curling stone in the *i*^th^ camera has not be assigned a GCID and meet the matching elimination criteria, we remove the matching relationship in bipartite graph.

By the elimination criteria, the solution space of the bipartite graph matching is greatly reduced. Then, we find the optimal assignment solution by Hungarian algorithm. With the long-term matching mechanism, the region growing algorithm can be executed along the time dimension and curling stones which have been occluded for a long time can find the corresponding ID.

For these curling stones which have not been assigned the GCID by LSTMM-based region growing, we assign new GCIDs to newly appearing curling stones. Because of the incremental ID allocation strategy, we can detect the legality of appearing curling stone which can help us largely eliminate false detections. For each camera, the new curling stones' GCID information, which are potential curling seeds for regional growing algorithm, can also be passed across frames.

Finally, curling stones are merged from different cameras which have a same global curling ID. To improve the accuracy of multicamera sensor fusion, we use the accuracy confidence scores as a guide for weighted fusion of curling coordinates. The accuracy confidence score of each curling stone in different cameras can be learned by convolution neural network. It can be calculated by

(20) $$\begin{array}{c} c_{t}^{m}=\frac{\sum_{i=1}^{N}w_{ij}c_{t}^{ij}}{\sum_{i=1}^{N}w_{ij}}, \end{array}$$

where all the merging curling stones *c*_*t*_^*ij*^ have the same GCID across cameras and *N* is the number of cameras that the curling can be observed simultaneously at the moment. The accuracy confidence scores, denoted as *w*_*ij*_, can be calculated by equation [4]. Through multicamera sensor fusion, we get the real-time global trajectories of curling stones.

### 4.6. Motion Analysis and Trajectory Prediction

#### 4.6.1. Motion Analysis

High-frequency motion capture can help us to analyze the motion state of curling stone all the time, but measurement noise has a big impact on this problem, especially in multicamera sensor systems. We store the numerical values of *L*_*t*_ and *θ*_*t*_ at corresponding time *T*_*t*_, where *L*_*N*_ = *L*_*N*−1_+∆*l*. As shown in figure S9, *θ*_*t*_ can be solved by the angle between the curling handle and the *x* axis in the world coordinate system. Meanwhile, to reduce the influence of accumulated measurement errors of *L*_*t*_ which is perpendicular to the trajectory direction, we project the current curling position coordinates to the tangent direction of the curling trajectory. To approximate the motion of curling at time *T*_*t*_, we use asymmetric weighted least-square (AWLS) method along time domain to locally solve this problem. The sampling points can be approximated by

(21) $$\begin{array}{c} F^{\Omega_{T_{t}}}\left(t\right)=\overset{\mu }{\underset{i=1}{\sum }}p_{i}\left(t\right)q_{i}\left(t\right)=q^{T}\left(t\right)p\left(t\right),\forall t\in \Omega_{T_{t}}, \end{array}$$

where **q**^*T*^(*t*) is a *μ*-dimensional basis function vector, **p**(*t*) is a coefficient vector which needed to be estimated, *Ω*_*T*_*t*__ is an asymmetric local neighborhood [*T*_*t*−*t*_1__, *T*_*t*+*t*_2__] around time *T*_*t*_. To estimate the velocity and rotation of curling stone, we set *t*_1_ = 50 and *t*_2_ = 10 which can real-time output of results delayed by 10 frames. We set **q**(*t*) = [1, *t*, *t*^2^]^*T*^ with its order number *μ* = 3. The coefficient vector **p**(*t*) can be solved by minimize the following weighted least square errors:

(22) $$\begin{array}{c} {\left.E\right|}_{\Omega_{T_{t}}}=\overset{t+t_{2}}{\underset{T_{t}=t-t_{1}}{\sum }}w_{T_{t}}\left[F^{T_{t}}\left(t\right)-g^{T_{t}}\right], \end{array}$$

where *g*^*T*_*t*_^ is the actual observed value. It can be formulated as a vector matrix formulation:

(23) $$\begin{array}{c} {\left.E\right|}_{\Omega_{T_{t}}}={\left(Qp–g\right)}^{T}W\left(Qp–g\right), \end{array}$$

where

(24) $$\begin{array}{c} Q=\left[\begin{array}{ccc} 1 & T_{t-t_{1}} & T_{t-t_{1}}^{2}\\ \vdots & \ddots & \vdots \\ 1 & \cdots T_{t+t_{2}} & T_{t+t_{2}}^{2} \end{array}\right], \end{array}$$

(25) $$\begin{array}{c} g=\left[g_{t-t_{1}},\cdots ,g_{t+t_{2}}\right], \end{array}$$

(26) $$\begin{array}{c} W=\left[\begin{array}{cccc} w_{T_{t-t_{1}}} & 0 & \cdots & 0\\ 0 & w_{T_{t-t_{1}+1}} & \cdots & 0\\ \vdots & \vdots & \ddots & \vdots \\ 0 & 0 & \cdots & w_{T_{t+t_{2}}} \end{array}\right]. \end{array}$$

By minimizing the error function, we can obtain the coefficient vector **p** = (*p*_1_, *p*_2_, *p*_3_) by

(27) $$\begin{array}{c} p={\left(Q^{T}WQ\right)}^{-1}Q^{T}Wg. \end{array}$$

We can also get the velocity and acceleration by the first derivative and the second of *F*^*Ω*_*T*_*t*__^(*t*) with respect to *t*.

The optimizations process for curling stone's angle *θ*_*t*_ and distances *L*_*t*_ are slightly different in terms of weights. For the fitting problem of *L*^*Ω*_*T*_*t*__^(*t*), the weight at *T*_t__*i*_ is caculated by

(28) $$\begin{array}{c} {\left.{\text{w}}_{{{\text{T}}_{\text{t}}}_{i}}\right|}_{L}=\frac{1}{\sqrt{2\pi }\sigma_{t}}\;e^{\left(-{\left({T_{t}}_{i}-T_{t}\right)}^{2}\right)/2\sigma_{t}^{2}}\frac{1}{\sqrt{2\pi }\sigma_{v}}\;e^{\left(-{\left({V_{t}}_{i}-V_{t}\right)}^{2}\right)/2\sigma_{v}^{2}}, \end{array}$$

where *T*_*t*__*i*_ ∈ *Ω*_*T*_*t*__, *σ*_*t*_ is the time distance variance, and *σ*_*t*_ is the velocity distance variance. The temporary speeds *V*_*t*_ is given by

(29) $$\begin{array}{c} V_{t}=\frac{L_{t}-L_{t-1}}{T_{t}-T_{t-1}}. \end{array}$$

For *θ*^*Ω*_*T*_*t*__^(*t*) function estimation, the weight at *T*_*t*__*i*_ is caculated by

(30) $$\begin{array}{c} {\left.{\text{w}}_{{T_{t}}_{i}}\right|}_{\theta }=\frac{1}{\sqrt{2\pi }\sigma_{t}}\;e^{\left(-{\left({T_{t}}_{i}-T_{t}\right)}^{2}\right)/2\sigma_{t}^{2}}\frac{1}{\sqrt{2\pi }\sigma_{w}}\;e^{\left(-{\left({W_{t}}_{i}-W_{t}\right)}^{2}\right)/2\sigma_{w}^{2}}, \end{array}$$

where *σ*_*w*_ denotes the angular velocity variance. The temporary speeds *W*_*t*_ is given by

(31) $$\begin{array}{c} W_{t}=\frac{\theta_{t}-\theta_{t-1}}{T_{t}-T_{t-1}}. \end{array}$$

By using the asymmetric weighted least-square (AWLS), we can robust estimate the velocity, acceleration, and angular velocity of curling stone in real time, where these data can reflect the quality of the ice surface and help athlete train.

#### 4.6.2. Trajectory Prediction

The properties of the ice surface change with temperature, humidity, etc. To simply this problem, we assume that the overall performance of the ice remains constant during each throw; therefore, the motion of curling can approximately satisfy the Markov assumption which we can adopt sequence model to predict the curling stone's trajectory.

The long short-term memory (LSTM) [32] network which is a variant of recurrent neural networks has been proven to be very successful for sequence prediction task [35–37] such as speech recognition, machine translation, and human trajectory prediction. We introduce an encoder-decoder framework based on LSTM which predicts the future curling stone's trajectory *Y* = {*Y*_0_, *Y*_1_, ⋯, *Y*_pred_} based on curling stone's spatial observation *X* = {*X*_0_, *X*_1_, ⋯, *X*_obs_} in a throw. The framework of our trajectory prediction model is shown in Figure 2(c). Our model consists of three key components: encoder, rotation fusion module, and decoder. The encoder learns the physical properties of the ice surface and the motion pattern of curling stones from partial observations∆*X* = {∆*X*_0_, ∆*X*_1_, ⋯, ∆*X*_obs_}. Firstly, we use multilayer perceptron (MLP) to get the fixed length spatial embedding *e*_*i*_ of relative motion pattern ∆*X*_*i*_ = *X*_*i*_ − *X*_*i*−1_. Then, spatial embedding can be uesd as input by a LSTM cell of the encoder. We define the *i*^th^ observation as *X*_*i*_ = (*x*_*i*_, *y*_*i*_), the *j*^th^ prediction as *Y*_*j*_ = (*x*_*j*_, *y*_*j*_). The encoder at *i*^th^ observation can be defined as follows:

(32) $$\begin{array}{c} e_{i}=\psi \left(\Delta x_{i},\Delta y_{i},W_{ee}\right), \end{array}$$

(33) $$\begin{array}{c} h_{i}^{e}=\text{LSTM}\left(h_{i-1}^{e},e_{i-1},W_{\text{encoder}}\right), \end{array}$$

where *ψ*(·) is the spatial embedding function, *W*_*ee*_ is the embedding weight, and *W*_encoder_ is the weight of LSTM cell.

The trajectory of curling stone is close to a straight line when it is just thrown. The lateral movement of the curling stone caused by the rotation is even smaller than the measurement error of the system. The rotation angle is extracted from the handle landmarks of the curling stone, which is a local feature of the curling stone and is easily affected by the occlusion of the curling stone. Therefore, we use the rotation direction instead of the rotation angle to obtain a more robust evaluation. Therefore, we design a rotation fusion module to merge the rotation direction information into observation hidden state to get the hidden state *h*_obs_.

To keep trajectory prediction consistent with past trajectory observation during a throw, we initialize the state of decoder by *h*_obs_, where the hidden state *h*_obs_ contains the assessment of the ice surface at this casting. The decoder at *j*^th^ observation can be defined as follows:

(34) $$\begin{array}{c} e_{j}=\phi \left(\Delta x_{j-1},\Delta y_{j-1},W_{ed}\right), \end{array}$$

(35) $$\begin{array}{c} h_{j}^{d}=\text{LSTM}\left(h_{j-1}^{d},e_{j},W_{\text{decoder}}\right), \end{array}$$

(36) $$\begin{array}{c} \Delta X_{j}=\varphi \left(h_{j}^{d}\right), \end{array}$$

where *ϕ*(·) is the spatial embedding function in decoder, *W*_*ed*_ is the embedding weight, *W*_decoder_ is the weight of LSTM cell in decoder, and *φ*(·) is the MLP function.

We embed the coordinate as a 16-dimensional vector. The dimensions of the hidden state for encoder and decoder are 32. We train the model by minimizing *L*_2_ loss by an Adam optimizer, which can minimize the deviation of the predicted trajectory from the actual ground truth. In the inference stage, we can predict the trajectory by partial observation of a whole trajectory, the prediction result can maintain the same motion patterns as observation in the same ice surface conditions.

### 4.7. Performance Tests

All the tests were conducted on the Intel(R) Xeon(R) Gold 6226R CPU @ 2.90 GHz and Tesla T4 GPU.

#### 4.7.1. Runtime Tests

A video with ten targets was used for testing performance of the single-camera tracking module. RetinaNet [21] with multiscale feature pyramids network (FPN) [22] was used to generate initial LTTP firstly, and then, the refine module was used to improve the quality of the LTTP. The input image was resized by our system from 2160 × 3840 to 864 × 1536, where the size of target was 32 × 32, because it was time consuming if the full image was detected while the targets in the image were small. To speed up the runtime of the refine module and curling landmark detection on refined LTTP, we took multiple targets patch from single view as a same batch input and feed into the network. In the refine module, the target patch was cropped from the original image and the ratio of the target in the patch was fixed as 1.2. The initial LTTP was resized to 112 × 112, so as to obtain the refined LTTP. In the landmark detection, the target was cropped from the refined LTTP where the proportion of curling stone was 1.1. Similarly, the LTTP was resized to 112 × 112 and then fed into the landmark detection network. As shown in table S2, the process took about 7.598 ms, where detection took 6 ms, the refine module took 0.839 ms, and the landmark detection took 0.759 ms. In the tracking stage, the refined LTTP associated the spatial temporal information across frames, where the proportion of the target in template patch and search patch was about 1/2 and 1/3, respectively. The template branch encoded the historical refined LTTP which was resized to 63 × 63, and the detect branch encoded the search patch which was resized to 95 × 95 in current frame. Then, the refine module was used to improve quality of the LTTP and avoid false detections. After that, we cropped the target from the refined LTTP and fed the local target patch into the landmark detection network. As shown in table S3, the whole process took 5.169 ms, including 3.571 ms to update the LTTP, 0.839 ms to improve quality of the LTTP, and 0.759 ms to detect landmarks of curling. Our distortion model was a model from the distortion image to the rectified image, resulting that the image point could be corrected quickly in 0.001 ms without iterative distortion correction.

For multicamera tracking module, we used all the cameras of a single track for offline testing, which consisted of 12 cameras. We set dist_thr_ = 300 mm, *v*_thr_ = 3 m/s, while limiting the region allocated by GCID where the newly appearing curling could only appear in the starting area of the curling throw. The cross-camera data association for 12 cameras took 0.1711 ms.

To test the overall performance of CurlingHunter in real world, we used multithreading to test, where each video was processed in parallel using one thread and the cross-camera tracking used a single thread. In order to enhance the utilization of the graphics card, we used Tesla T4 GPU to process the data of three videos at the same time. We combined detection, tracking, and the refine module where the detection interval was 10 frames. Although the number of curling stones that appeared at different times and different cameras was different, the overall time overhead of CurlingHunter maintained at only ~9.005 ms per batch of video frames. In a word, the millisecond-level processing speed paves the way for real-time applications of CurlingHunter in curling games.

#### 4.7.2. Motion Tests

We evaluated the results of speed and angle measurements in a wheelchair race where no athletes rubbed the ice. To reduce the influence of accumulated measurement errors which was perpendicular to the trajectory direction, we projected the current curling stone's position coordinates to the tangent direction of the curling stone's trajectory. At the same time, we used asymmetric weighted least-square (AWLS) method along time domain to locally solve this problem where we used the next 10 frames motion information and historical 50 frames motion information to smooth the velocity of the current frame. We set *σ*_*t*_ = 1.2 s and *σ*_*v*_ = 0.5 m/s, as shown in Figure 3(a), the noise in the velocity calculation due to measurement errors was be eliminated in real time. As shown in Figure 3(b), angular smoothing was similar to speed smoothing, where we set *σ*_*t*_ = 1.2 s and *σ*_*w*_ = 0.3 rad/s.

#### 4.7.3. Trajectory Prediction Tests

We collected a portion of the curling motion data measured by the multicamera system and randomly divided the training set and the validation set according to a certain proportion. To verify the validity of our modeling of curling stone's trajectory prediction, we used a 3-second motion pattern of curling stone to predict the trajectory of curling stone in the next 9 seconds, where the overall state of the ice surface and the motion pattern of curling stone could be estimated approximately from 3-second observational trajectory. The hidden state encoded by LSTM could encode the motion pattern of the curling stone to help us predict curling stone's trajectory in the future. Meanwhile, we designed a rotation fusion module to enhance the effect of rotation on future trajectory prediction. We calculated the cumulative distance error of the curling trajectory. As shown in Figure 3(g), our method predicts future trajectories better than those estimated by Kalman filtering. Among them, the error of curling stone's trajectory prediction mainly comes from the measurement error of the observation trajectory and the uneven distribution state of the ice surface.

## Data Availability

All data are available in the main text or the supplementary materials.

## Supplementary Materials

Supplementary Materials Figure S1: the size of the curling ice tracks. Figure S2: positions and layouts of forty-two cameras. Figure S3: jurisdictional areas of forty-two cameras. Figure S4: actual monitor areas of 12 cameras in F2. Figure S5: actual monitor areas of 22 cameras in CW. Figure S6: actual monitor areas of 8 cameras in Truss. Figure S7: red curling stone and yellow curling stone in ice track. Figure S8: the examples of lens distortion correction. Figure S9: multicamera velocity analysis. Figure S10: motion analysis. Figure S11: motion analysis of curling games in real time. Figure S12: the management system. Table S1: LSTMM. Table S2: ablation experiments of the refine module. Table S3: runtime tests of single-camera detection process. Table S4: runtime tests of single-camera tracking process. Table S5: comparison of existing AI systems for sports and CurlingHunter. Movie S1: restoring the actual trajectory to the track so as to verify the accuracy of CurlingHunter. Movie S2: performance test of CurlingHunter. Movie S3: the broadcast live of CurlingHunter in 2022 Beijing Winter Olympics. Movie S4: the broadcast live of CurlingHunter in 2022 Beijing Winter Paralympics. Movie S5: motion analysis.
