# Supplementary material for: An AI-Based Curling Game System for Winter Olympics
Source: Research (Wash D C). 2022 Oct 20;2022:9805054. doi: 10.34133/2022/9805054 (PMC9639444; doi:10.34133/2022/9805054)
Supplement: Supplementary Materials — Figure S1: the size of the curling ice tracks. Figure S2: positions and layouts of forty-two cameras. Figure S3: jurisdictional areas of forty-two cameras. Figure S4: actual monitor areas of 12 cameras in F2. Figure S5: actual monitor areas of 22 cameras in CW. Figure S6: actual monitor areas of 8 cameras in Truss. Figure S7: red curling stone and yellow curling stone in ice track. Figure S8: the examples of lens distortion correction. Figure S9: multicamera velocity analysis. Figure S10: motion analysis. Figure S11: motion analysis of curling games in real time. Figure S12: the management system. Table S1: LSTMM. Table S2: ablation experiments of the refine module. Table S3: runtime tests of single-camera detection process. Table S4: runtime tests of single-camera tracking process. Table S5: comparison of existing AI systems for sports and CurlingHunter. Movie S1: restoring the actual trajectory to the track so as to verify the accuracy of CurlingHunter. Movie S2: performance test of CurlingHunter. Movie S3: the broadcast live of CurlingHunter in 2022 Beijing Winter Olympics. Movie S4: the broadcast live of CurlingHunter in 2022 Beijing Winter Paralympics. Movie S5: motion analysis. [file 9805054.f1.zip › 9805054.f1/Supplementary Materials.docx]

Supplementary Materials for

**An AI-based curling game system for Winter Olympics**

Xuanke Shi, Quan Wang, Chao Wang, Rui Wang, Longshu Zheng, Chen Qian, Wei Tang*

Correspondence to: weitang@zju.edu.cn

**This PDF file includes:**

Figure S1. The size of the curling ice tracks.

Figure S2. Positions and layouts of forty-two cameras.

Figure S3. Jurisdictional areas of forty-two cameras.

Figure S4. Actual monitor areas of 12 cameras in F2.

Figure S5. Actual monitor areas of 22 cameras in CW.

Figure S6. Actual monitor areas of 8 cameras in Truss.

Figure S7. Red curling stone and yellow curling stone in ice track.

Figure S8. The examples of lens distortion correction.

Figure S9. Muticamera velocity analysis.

Figure S10. Motion analysis.

Figure S11. Motion analysis of curling games in real time.

Figure S12. The management system.

Table S1. LSTMM.

Table S2. Ablation experiments of the Refine Module.

Table S3. Runtime tests of single-camera detection process.

Table S4. Runtime tests of single-camera tracking process.

Table S5. Comparison of existing AI systems for sports and CurlingHunter.

Movie S1. Restoring the actual trajectory to the track so as to verify the accuracy of CurlingHunter.

Movie S2. Performance test of CurlingHunter.

Movie S3. The broadcast live of CurlingHunter in 2022 Beijing Winter Olympics.

Movie S4. The broadcast live of CurlingHunter in 2022 Beijing Winter Paralympics.

Movie S5. Motion analysis.

**Other Supplementary Materials for this manuscript include the following:**

Movies S1 to S5


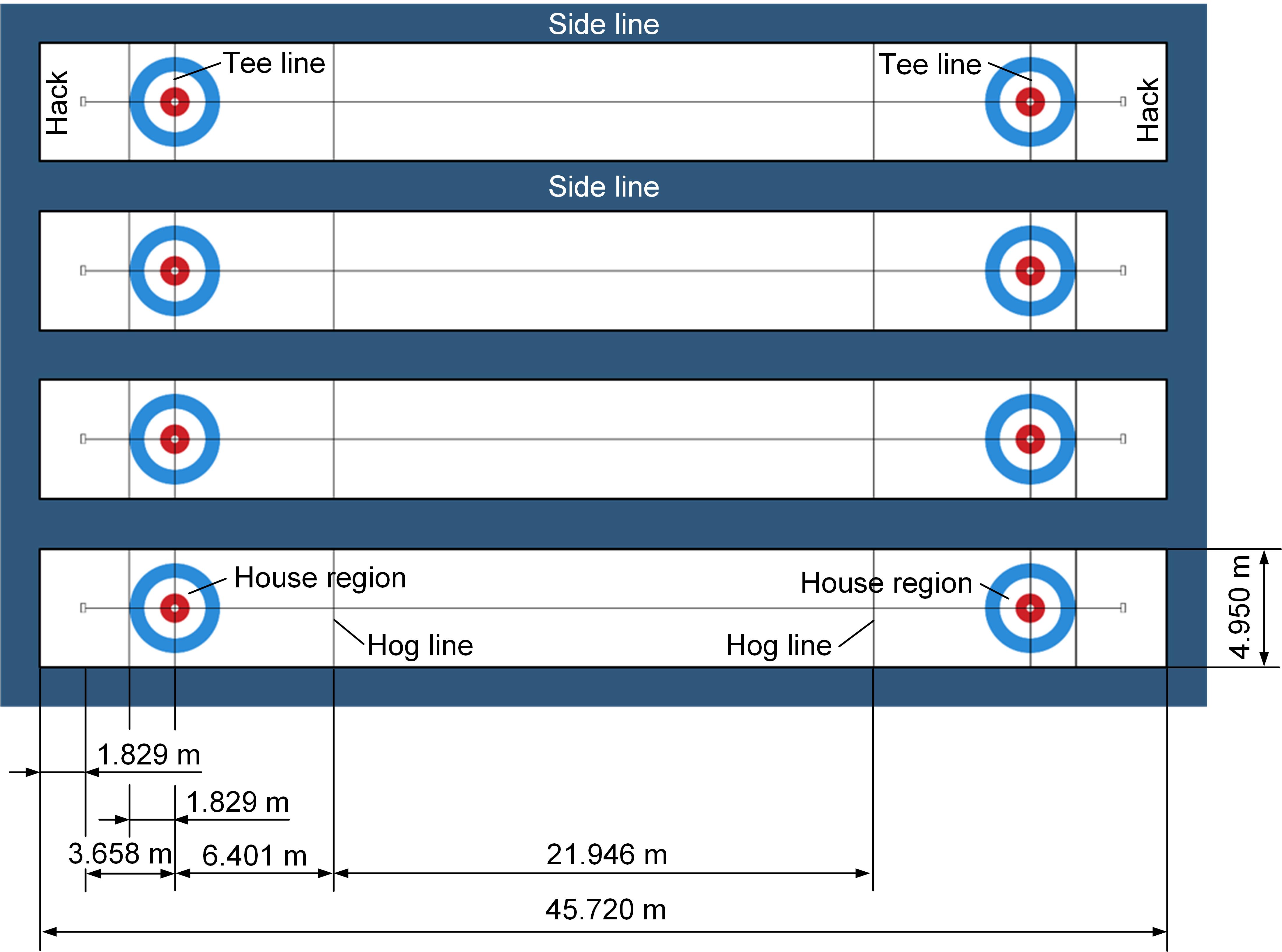


**Figure S1. The size of the curling ice tracks.** There are four ice tracks in curling game, where each ice track consists of side line, house region, hack, tee line, and hog line.


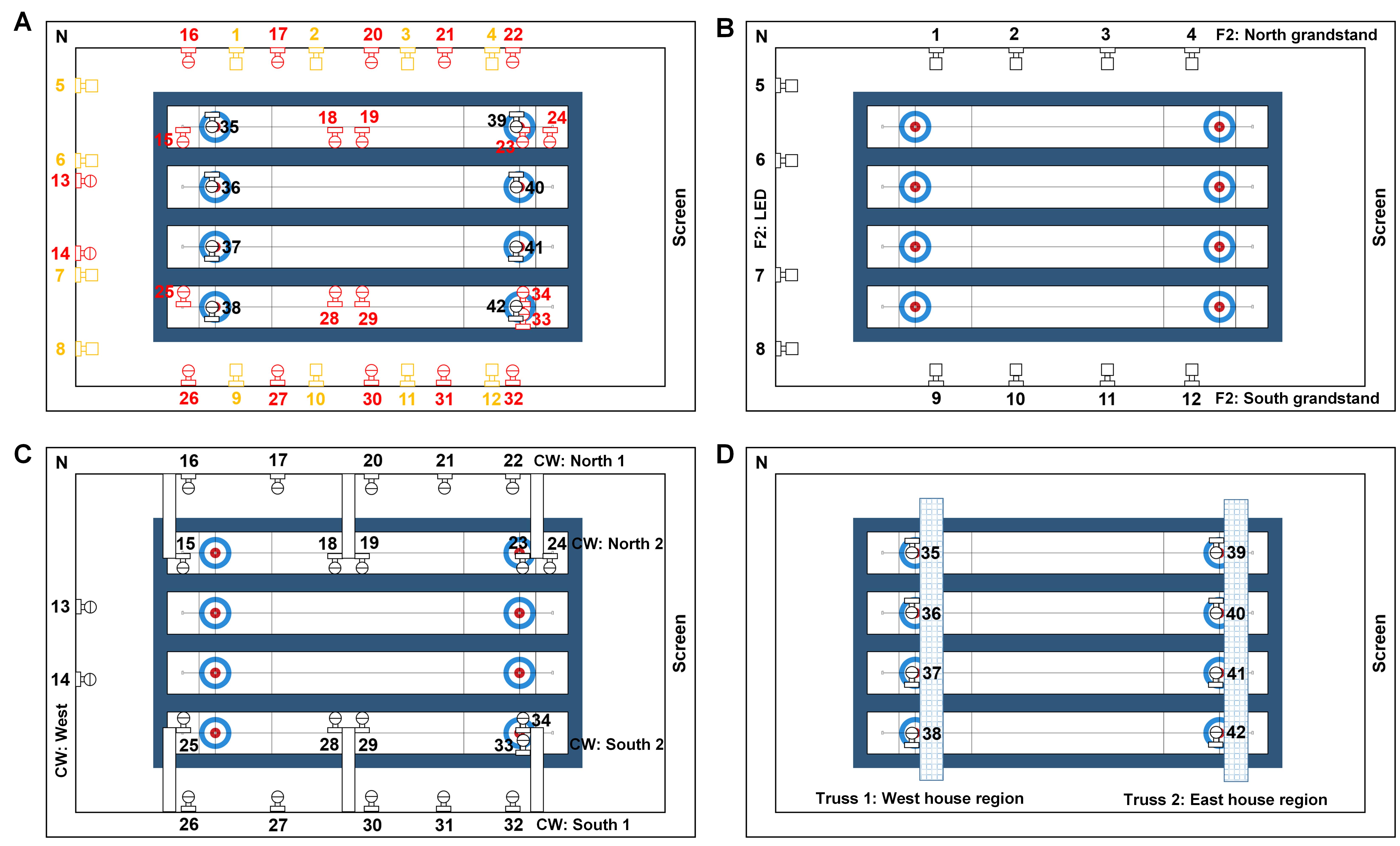


**Figure S2. Positions and layouts of forty-two cameras.** (**A**), Forty-two cameras were divided into three heights, i.e. 2nd floor of grandstand (F2), Cat walk (CW), and Truss. (**B**), Positions and layouts of box cameras 1-12 in F2. (**C**), Positions and layouts of speed dome cameras 13-34 in CW. (**D**), Positions and layouts of speed dome cameras 35-42 in Truss.


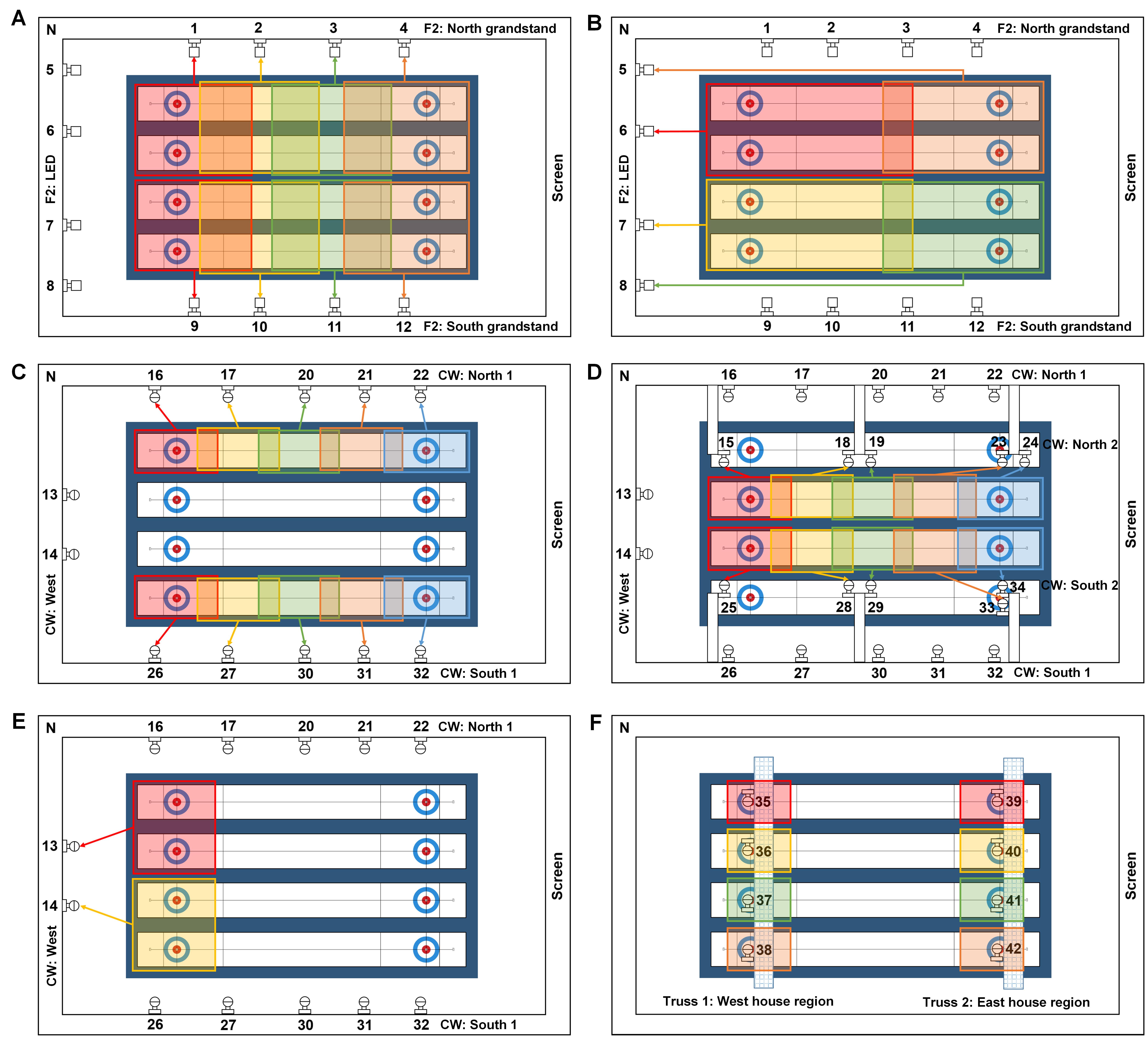


**Figure S3. Jurisdictional areas of forty-two cameras.** (**A**), Jurisdictional areas of cameras 1-4 and 9-12 in F2. (**B**), Jurisdictional areas of cameras 5-8 in F2. (**C**), Jurisdictional areas of cameras 16, 17, 20-22 and 26, 27, 30-32 in CW in F2. (**D**), Jurisdictional areas of cameras 15, 18, 19, 23, 24 and 25, 28, 29, 33, 34 in CW. (**E**), Jurisdictional areas of cameras 13, 14 in CW. (**F**), Jurisdictional areas of cameras 35-42 in Truss.


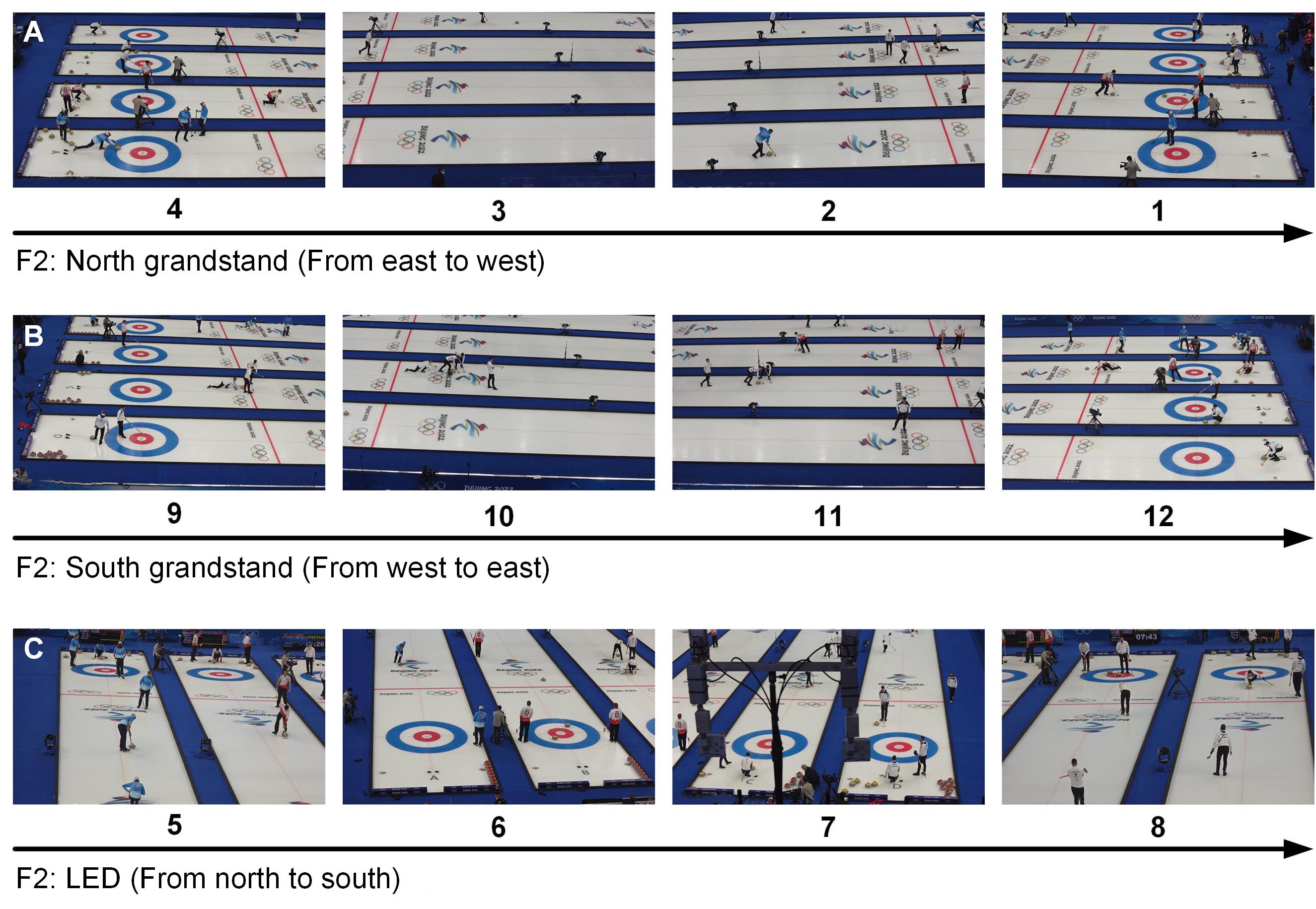


**Figure S4. Actual monitor areas of 12 cameras in F2.** (**A**), Actual monitor areas of cameras 1-4 in North grandstand. (**B**), Actual monitor areas of cameras 9-12 in South grandstand. (**C**), Actual monitor areas of cameras 5-8 in LED.


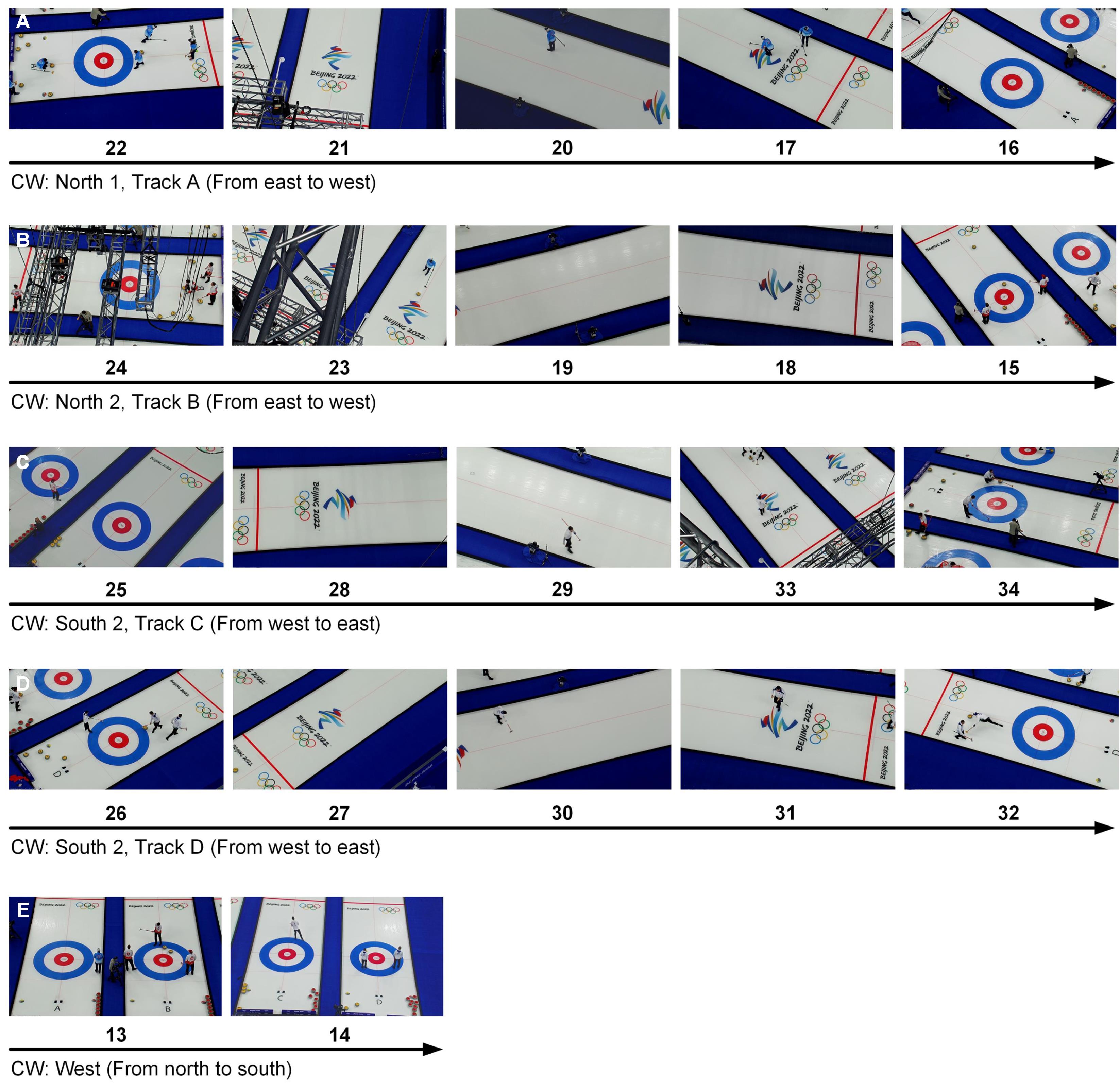


**Figure S5. Actual monitor areas of 22 cameras in CW.** (**A**), Actual monitor areas of cameras 16, 17, 20-22 in North 1. (**B**), Actual monitor areas of cameras 15, 18, 19, 23, 24 in North 2. (**C**), Actual monitor areas of cameras 25, 28, 29, 33, 34 in South 2. (**D**), Actual monitor areas of cameras 26, 27, 30-32 in South 1. (**E**), Actual monitor areas of cameras 13, 14 in West.


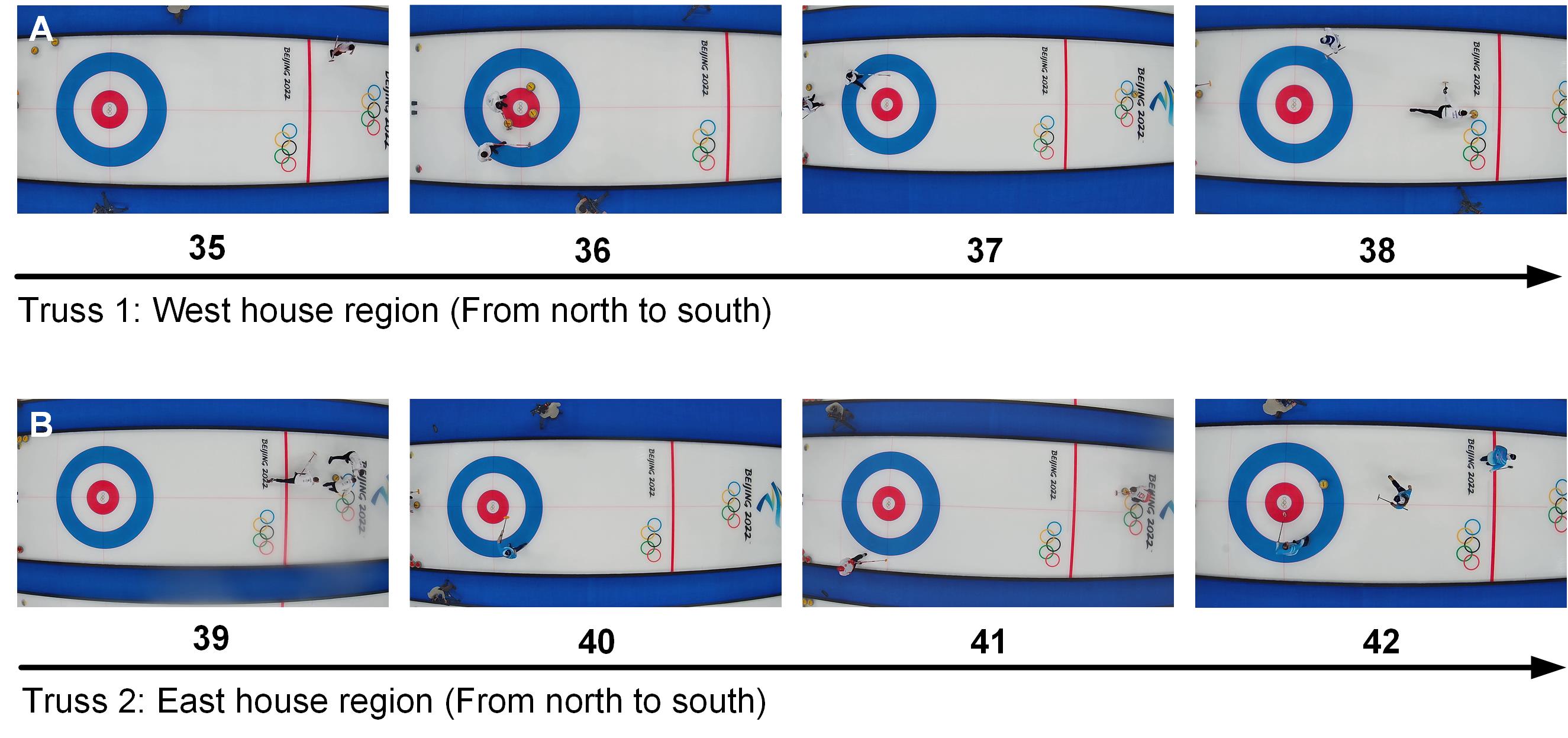


**Figure S6. Actual monitor areas of 8 cameras in Truss.** (**A**), Actual monitor areas of cameras 35-38 in Truss 1. (**B**), Actual monitor areas of cameras 39-42 in Truss 2.


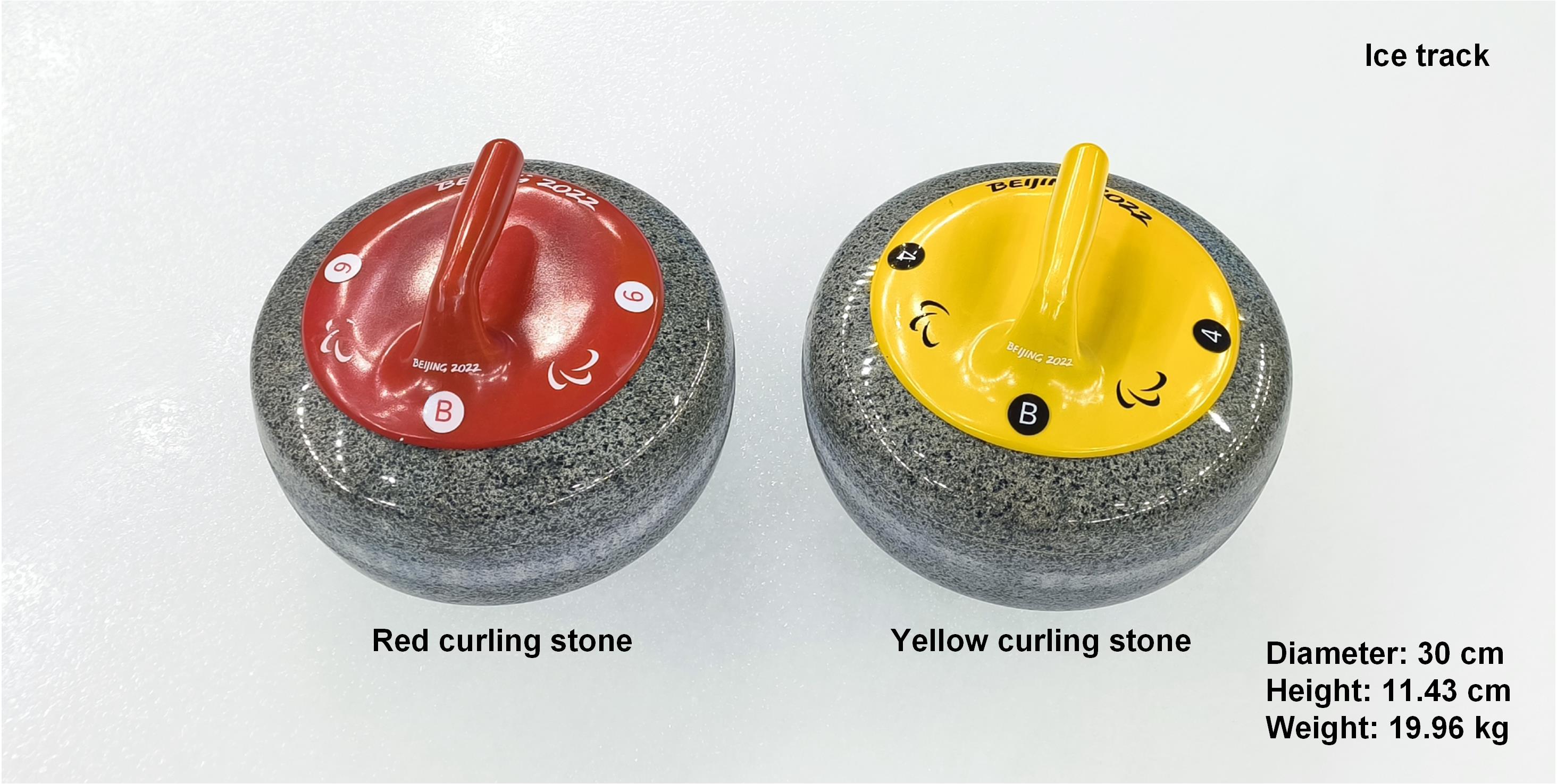


**Figure S7. Red curling stone and yellow curling stone in ice track.** The curling stones with same color have an identical appearance feature. The diameter, height, and weight of curling stone are 30 cm, 11.43 cm, and 19.96 kg, respectively.


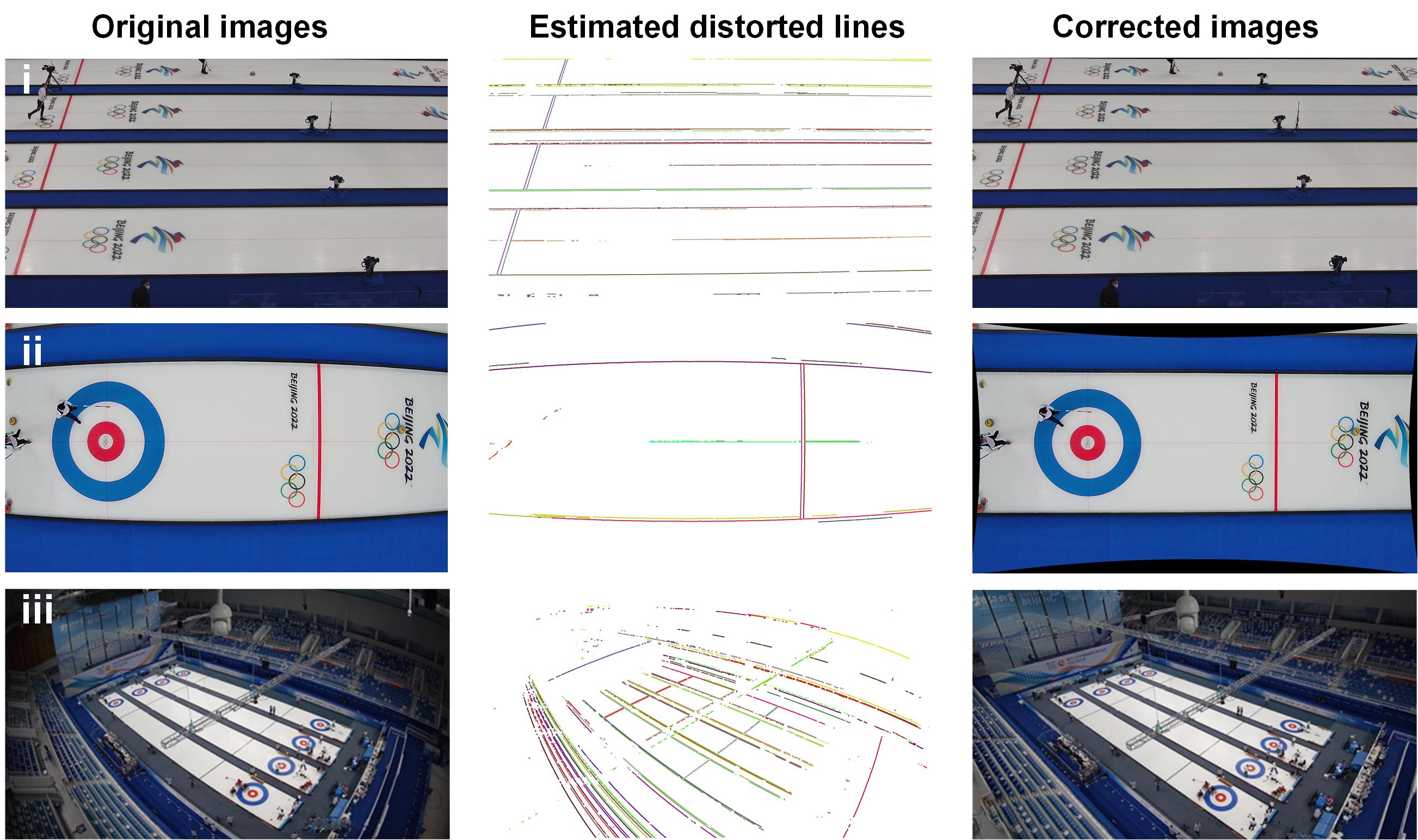


**Figure S8. The examples of lens distortion correction.**


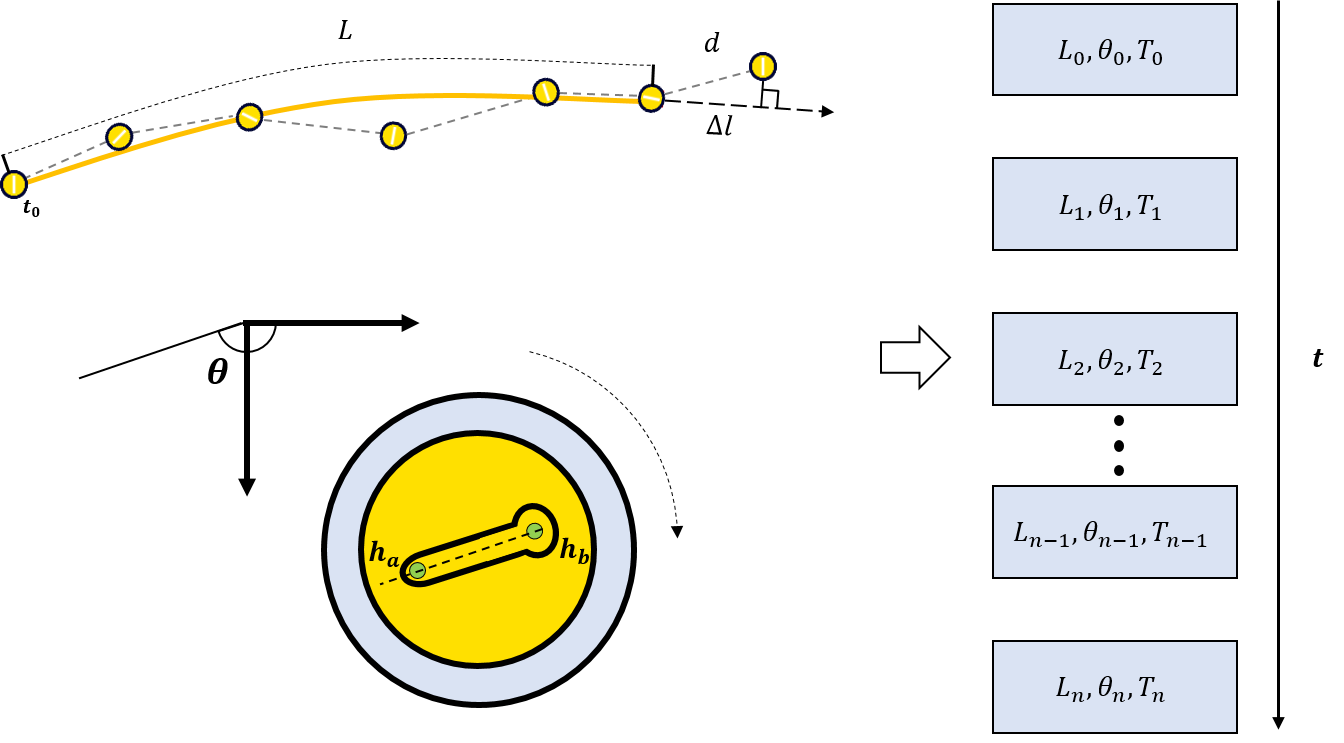


**Figure S9. Muticamera velocity analysis.** We project the current curling position coordinates to the tangent direction of the curling stone' trajectory to get the velocity. The rotation angle can be solved by curling landmarks.


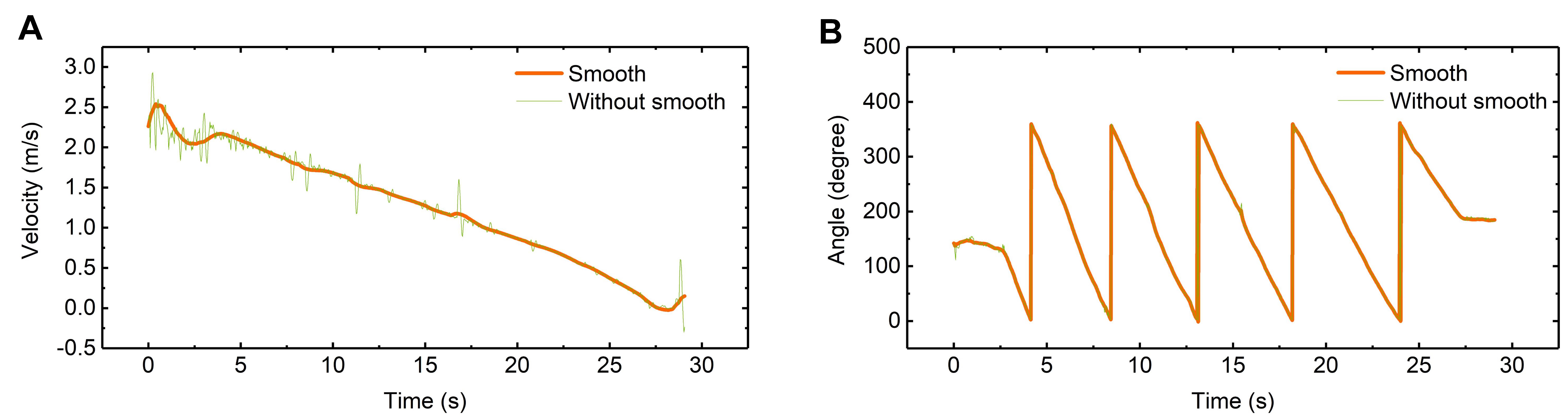


**Figure S10. Motion analysis.** (**A**), Real-time velocity smoothing. (**B**), Real-time angle smoothing.


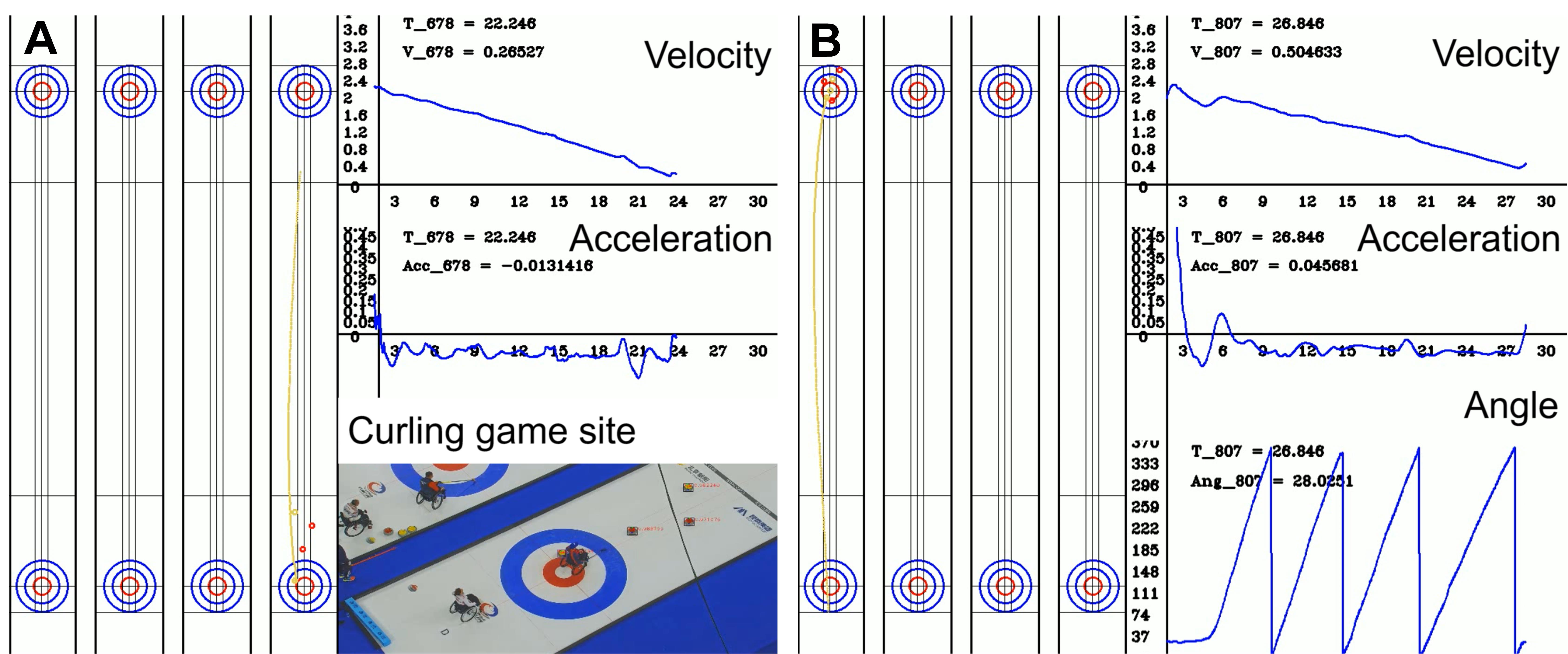


**Figure S11. Motion analysis of curling games in real time.** (**A**), Velocity, acceleration, curling game site and trajectory in real time. (**B**), Velocity, acceleration, angle and trajectory in real time.


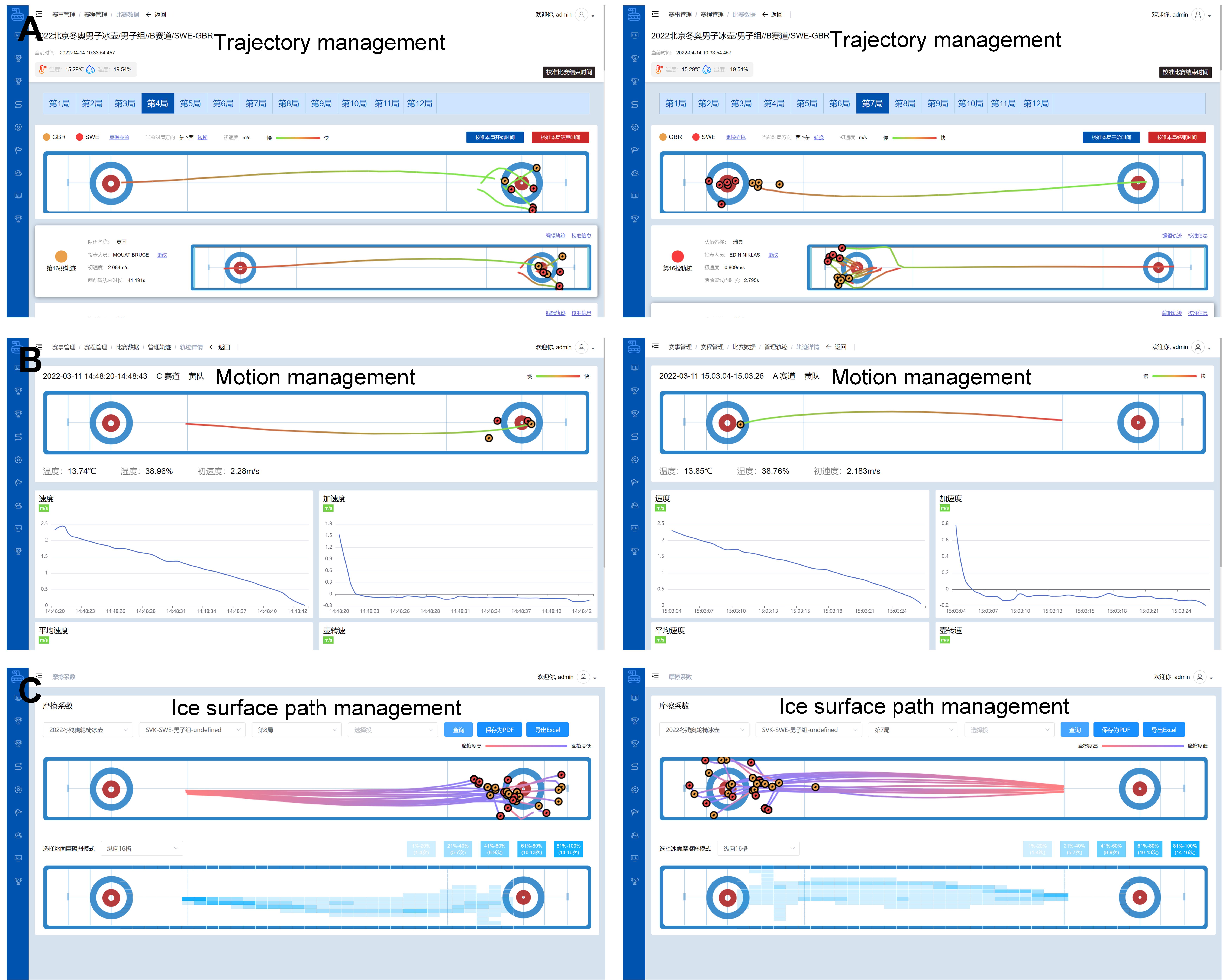


**Figure S12. The management system.** (**A**), The trajectory management system. (**B**), The motion analysis management system. (**C**), The ice surface path management system.

**Table S1. LSTMM**

|  | **Algorithm 1:** The proposed LSTMM Based Region Growing algorithm |
| --- | --- |
|  | **Input:** Aligned local trajectory set $\tilde{G_{t}}$ from N cameras. |
|  | **Output:** Global trajectory $F_{t}=\{{\left( F_{t}^{1}, F_{t}^{2}, \ldots, F_{t}^{M} \right), T}_{t} \}$ about M curling stones trajectory at aligned time $T_{t}$. |
| 1: | **for** each $\left( \tilde{G}_{t}^{i}, T_{t} \right) \in\tilde{G_{t}}$ do |
| 2: | Generate the curling seeds $S_{t}$ in $\tilde{G}_{t}^{i}$ which have been assigned GCID and can be tracked using single camera.//generate seeds |
| 3: | **end** |
| 4: | **for** each $\left( \tilde{G}_{t}^{i}, T_{t} \right)\in\tilde{G_{t}}$ **do** |
| 5: | **for** each curling $c_{t}^{ij}\in\tilde{G}_{t}^{i}$ **do** |
| 6: | region growing cross camera from $c_{t}^{ij}$ to seeds curling $c_{t}^{k}\in S_{t}$ and assigned GCID. // short-term matching machinism |
| 7: | **end** |
| 8: | region growing by temproal bigraph matching from curlings which haven’t assigned GCID in $\tilde{G}_{t}^{i},$ to global curlin history trajectories. //long-term matching machinism |
| 9: | **for** each curling $c_{t}^{j}\in\tilde{G}_{t}^{i}$ **do** |
| 10: | assign GCID for new appearing curling. // greedy assign |
| 11: | **end** |
| 12: | **end** |
|  | Merging the curling coordinates from same region with identical GCID and get Global trajectory $F_{t}$ |

**Table S2. Ablation experiments of the Refine Module**

| **Method** | **IDF1 ↑** | **MOTA ↑** | **MOTP ↓** |
| --- | --- | --- | --- |
| None | 74.5% | 69.3% | 0.137 |
| Regression Branch | 79.4% | 79.0% | 0.084 |
| Confidence Branch | 79.7% | 93.2% | 0.124 |
| **Refine Module** | **81.1%** | **94.5%** | **0.081** |

**Table S3. Runtime tests of single-camera detection process**

| **Submodule** | **Time (ms)** |
| --- | --- |
| RetinaNet | 6 |
| Refine Module | 0.839 |
| Landmark detection | 0.759 |
| Total of detection | 7.598 |

**Table S4. Runtime tests of single-camera tracking process**

| **Submodule** | **Time (ms)** |
| --- | --- |
| Template Branch | 0.903 |
| Detect Branch | 2.668 |
| Refine Module | 0.839 |
| Landmark detection | 0.759 |
| Total of tracking | 5.169 |

**Table S5. Comparison of existing AI systems for sports and CurlingHunter**

| **AI systems** | **Sports** | **Functions** | **Real time** |
| --- | --- | --- | --- |
| Hawk-Eye System [1] | Tennis | Assisting the referees in judging the games | NO |
| SportVU [2]  Stats Perform [2]  AutoStats [2] | Basketball (NBA)  Football  Baseball | Capturing the actions of the athletes | NO |
| Metrica [3] | Football | Tracking the ball in playback video | NO |
| SkillCorner [4] | Football | Tracking players, balls and referees based on live videos and then converting to a 2D version of the games | NO |
| Synergy [5] | Basketball (NBA, NCAAA)  Baseball | Capturing the actions of the athletes | NO |
| Smart Court [6] | Basketball (NBA)  Tennis (USTA)  Football | Recording actions, running distances, positions, reaction times of players from different views | NO |
| ShotTracker [7] | Basketball | Helping players improve their shooting skills | NO |
| Second Spectrum [8] | Basketball  Football | Reading the game strategy | NO |
| POSE [9] | Basketball | Assessing players' potential | NO |
| **CurlingHunter** | **Curling** | **Assisting athletes to play games**  **Enhancing the interest of the game**  **Recording game information** | **YES** |

**Movie S1. Restoring the actual trajectory to the track so as to verify the accuracy of CurlingHunter.**

**Movie S2. Performance test of CurlingHunter.**

**Movie S3. The broadcast live of CurlingHunter in 2022 Beijing Winter Olympics.**

**Movie S4. The broadcast live of CurlingHunter in 2022 Beijing Winter Paralympics.**

**Movie S5. Motion analysis.**

**Supplementary References**

1. N. E. Owens, C. Harris, and C. Stennett, “Hawk-eye tennis system,” in 2003 international conference on visual information engineering VIE 2003, pp. 182–185, Guildford, UK, 2003.
2. “SportVU, Stats Perform, and AutoStats,” Available at https://www.statsperform.com/.
3. “Metrica,” Available at https://metrica-sports.com/.
4. “SkillCorner,” Available at https://www.skillcorner.com/.
5. “Synergy,” Available at https://synergysports.com/.
6. “Smart Court,” Available at https://playsight.com/.
7. “ShotTracker,” Available at https://shottracker.com/.
8. “Second Spectrum,” Available at https://www.secondspectrum.com/.
9. “POSE,” Available at https://www.xenonstack.com/.
